# E2F-mediated activation of mTORC1 through the ubiquitin-proteasome system promotes lung adenocarcinoma progression

**DOI:** 10.1038/s41419-026-08863-2

**Published:** 2026-05-19

**Authors:** Wentao Xue, Mengen Wang, Tong Hu, Shina Guo, Anchi Hu, Yan Gu, Hongchang Wang, Guang Mu, Wenhao Zhang, Chenghao Fu, Zizhang Guo, Ke Wei, Yanan Li, Jun Wang

**Affiliations:** 1 Department of Thoracic Surgery, The First Affiliated Hospital of Nanjing Medical University, Nanjing, China; 2 Department of Oncology, The First Affiliated Hospital of Nanjing Medical University, Nanjing, China; 3 Department of Thoracic Surgery, Peking University People’s Hospital, Beijing, China; 4 Thoracic Oncology Institute, Peking University People’s Hospital, Beijing, China; 5 Department of Thoracic Surgery, Taihe Hospital, Shiyan, China; 6 School of Food Science and Pharmaceutical Engineering, State Key Laboratory of Microbial Technology, Nanjing Normal University, Nanjing, China

**Keywords:** Oncogenes, Non-small-cell lung cancer, Cancer epigenetics, Ubiquitin ligases, Ubiquitins

## Abstract

Lung adenocarcinoma (LUAD) is the major histological subtype of non-small cell lung cancer (NSCLC). The mechanistic target of rapamycin complex 1 (mTORC1), a master regulator of anabolic growth and metabolism, has been implicated in unfavorable prognosis and therapeutic resistance in LUAD. Nevertheless, how this association is mechanistically established remains insufficiently understood. Here, by integrating TCGA/GEO datasets with our institutional LUAD single-cell RNA-seq, we identified the atypical E2F factor E2F8 as the member most closely associated with the mTORC1 pathway and found that E2F8 is upregulated in LUAD and that higher E2F8 levels correlate with adverse clinicopathological features and poorer prognosis. In PC-9 and H1975 cells, E2F8 overexpression enhanced proliferation, clonogenicity, migration, and xenograft growth, whereas E2F8 silencing produced the opposite effects; rapamycin partially reversed these phenotypes, indicating mTORC1 dependence. Mechanistically, E2F8 activated transcription of the HECT-type E3 ligase WWP1, which recognized PPxY-containing TSC1 and mediated K48-linked polyubiquitination at K662, promoting proteasomal degradation of TSC1 and sustaining mTORC1 signaling, as evidenced by increased *p*-mTOR (Ser2448), *p*-S6K1, and *p*-4EBP1. WWP1 knockdown markedly blunted E2F8-induced mTORC1 activation, preserved TSC1 abundance, and attenuated downstream mTORC1 readouts under E2F8/WWP1 activation, supporting TSC1 as the critical substrate. Pharmacologic WWP1 inhibition with indole-3-carbinol (I3C) restored TSC1, reduced *p*-mTOR (Ser2448), and suppressed LUAD xenograft growth, defining an E2F8-WWP1-TSC1-mTORC1 axis as a targetable circuit in LUAD.

## Introduction

Globally, lung cancer accounts for the highest burden of cancer mortality [1], whose predominant subtype is lung adenocarcinoma (LUAD) [2]. Despite substantial advancements in driver gene-based targeted therapies and immunotherapies, a significant proportion of patients exhibit non-durable treatment responses and are susceptible to developing acquired resistance [3]. Pathways governing tumor growth and metabolism are closely linked to the development of therapeutic drug resistance in cancer [4, 5]. This underscores the need to identify novel druggable targets by examining the fundamental mechanisms that link tumor growth and metabolism.

The mechanistic target of rapamycin complex 1 (mTORC1), a known signaling hub from yeast to mammals, integrates nutrients and stress signals to sustain growth and metabolism in cells [6–8]. It promotes protein production, lipids and nucleotides, and suppresses autophagy and catabolism [6–8**]**. Dysregulation of mTORC1 signaling occurs frequently in human cancers and correlates significantly with tumor progression [9, 10]. Beyond occasional mTOR amplification, alterations in oncogenes and tumor suppressors—KRAS, EGFR/IGF1R, PIK3CA, AKT, PTEN, STK11/LKB1, TSC1, and TSC2—converge to activate and sustain mTORC1 signaling, maintaining pathway output even under adverse conditions and driving malignant growth and metabolic reprogramming [6, 8, 9, 11]. In LUAD, studies have shown that up to 90% of cases exhibit activation of the mTOR pathway [12]. Moreover, functional loss events in negative regulators such as LKB1 and p53 further relieve inhibition of mTORC1 [6, 13]. In LUAD, where EGFR and KRAS mutations are prevalent, recent studies suggest that pharmacologic inhibition of mTORC1 may help overcome therapeutic resistance [14, 15]. Therefore, understanding and intervening in the “gatekeeper” control of mTORC1 from a more upstream and plastic homeostasis level presents potential therapeutic advantages.

The E2F transcription factor family, which serves a pivotal function in diverse biological processes like DNA replication, apoptosis, and cell differentiation, features evolutionarily preserved DNA-binding domains [16, 17]. E2Fs act as a key regulatory axis for cell cycle-dependent gene expression [18], and elevated E2F activity or overexpression of its target genes in cancer correlates with poor prognosis [18, 19]. Similar to E2F7, E2F8 is an atypical E2F whose transcription is induced at the G1-S transition, remains elevated through S phase, and peaks around S-G2; as a target within the E2F network, it cooperates with E2F7 to balance the cell cycle [16]. Studies across multiple cancer types, including lung adenocarcinoma, have shown that E2F8 is linked to pro-tumorigenic phenotypes such as accelerated cell-cycle progression, enhanced EMT and angiogenesis, and modulation of apoptosis [20–24]. Furthermore, E2F8 is a transcriptional stimulator which increases transcription of genes related to tumors for further oncogenicity [24, 25]. However, the molecular connection between E2F8 and the growth- and metabolism-related mTORC1 pathway remains unclear and requires further elucidation.

WWP1 (WW domain E3 ubiquitin protein ligase 1) is an E3 ubiquitin protein ligase belonging to the HECT family, whose typical structure includes a C2 domain, tandem WW domains for substrate recognition, and a C-terminal HECT catalytic domain [26]. WWP1 targets LATS1, p27Kip1, AMPKα2 and PTEN proteins, and it promotes tumor growth with pro-oncogenic effects well known in many cancers, including lung cancer [27–30]. However, the upstream and downstream regulation of WWP1 in LUAD remains unexplored.

In this study, we identified E2F8 as the E2F family member most closely linked to mTORC1 signaling in LUAD and investigated whether it promotes LUAD progression through transcriptional upregulation of WWP1. We further examined whether WWP1-mediated destabilization of TSC1 contributes to mTORC1 activation and whether pharmacological inhibition of WWP1 could suppress this pathway and inhibit tumor growth. Together, these analyses were intended to define the E2F8-WWP1-TSC1-mTORC1 axis in LUAD and explore its therapeutic relevance.

## Methods and materials

### Cell culture

Human LUAD cell lines A549, NCI-H1299, NCI-H2087, NCI-H1975, PC-9, and A427, human embryonic kidney cell line HEK293T, and human bronchial epithelial cells BEAS-2B and 16-HBE were obtained from the American Type Culture Collection (ATCC). A549, BEAS-2B, 16-HBE, and HEK-293T cells were grown in Dulbecco’s Modified Eagle’s Medium (DMEM) with 10% fetal bovine serum. H1299, H2087, H1975, and PC-9 cells were cultured in RPMI-1640 with 10% FBS. MEM supplemented with 10% FBS was used for the culture of A427 cells. Cell lines were mycoplasma-free and kept at 37 °C with 5% CO_2_.

### Lung adenocarcinoma tissues collection and immunohistochemical assay (IHC)

LUAD tissues from human patients were collected during surgical procedures at the Department of Thoracic Surgery, Jiangsu Province Hospital. The research was sanctioned by the Ethics Committee of Jiangsu Province Hospital (Approval Number: 2024-SR-139), and all participants provided written consent.

Tissues were preserved in 10% neutral buffered formalin, embedded in paraffin and sectioned at 4 µm. Sections were baked, deparaffinized and rehydrated followed by heat induced antigen retrieval with citrate buffer (pH6.0). Peroxidase activity in the tissues was blocked, followed by overnight incubation with primary antibodies at 4 °C and subsequent incubation with HRP-conjugated secondary antibodies at room temperature. DAB staining was performed on the slides, which were then counterstained with hematoxylin. After rinsing the slides with appropriate buffer (e.g., PBS), they were dehydrated with graded ethanol series and mounted with neutral balsam. The IHC score was calculated as the product of staining intensity (0–3: none, weak, moderate, or strong) and the proportion of positive cells (0–4: 0%, 1–25%, 26–50%, 51–75%, or 76–100%), yielding a final score ranging from 0 to 12. Slides were independently evaluated by at least two blinded readers, and discrepant cases were jointly reviewed to reach a consensus score.

### RNA isolation, reverse transcription, and quantitative PCR (RT-qPCR)

Cells were isolated using FastPure Cell/Tissue Total RNA Isolation Kit (#RC113-01, Vazyme, China) and cDNA with HiScript II QRT SuperMix for qPCR (#R223-01, Vazyme, China) according to manufacturer’s instructions. Quantitative PCR was performed with gene specific primers (Table S2) using SYBR Green. After normalizing Ct values to the endogenous control, relative gene expression levels were measured using the 2^−ΔΔCt^ method.

### Cell transfection and lentiviral infection

Flag-E2F8, Flag-WWP1, Flag-WWP1-C890A and Myc-TSC1 were generated by inserting the relevant genes into the pCDH-CMV-MCS-EF1α-Puro vector. Plasmids containing HA-Ubiquitin wild-type (WT) and its variant forms (K6, K11, K27, K29, K33, K48, and K63) were sourced from Addgene. siRNAs targeting *E2F8* and *WWP1* are listed in Table S3. Lipofectamine 3000 (Invitrogen, USA) was utilized for transfection of the specified plasmids and siRNAs. Lentivirus particles carrying short hairpin RNA (shRNA) aimed at *E2F8* and its corresponding control were transfected into H1975 and PC-9 cell lines at a multiplicity of infection (MOI) of 10 over a 12-hour period. Following infection, cells were treated with 1 μg/mL puromycin for stable lines. Knockdown efficiency was validated via qPCR and western blot (WB).

### Assays for cell viability, proliferation, and migration

Cell Counting Kit-8 (CCK-8), 5-ethynyl-2′-deoxyuridine (EdU) incorporation, and colony formation assays were conducted following methods outlined in our prior publication [31].

For cell migration assays, 4 × 10^4^ serum-starved LUAD cells (PC-9 or H1975) suspended in 200 μL serum-free RPMI-1640 in the upper chambers of 24-well Transwell inserts with 8-μm pore size (Corning, NY, USA). The lower chambers contained 700 μL RPMI-1640 with 10% FBS as a chemoattractant. After 36 h of incubation at 37 °C, non-migrated cells on the upper membrane surface were removed with cotton swabs. Migrated cells on the lower surface were fixed with 4% paraformaldehyde (KGIHC016CS, KeyGEN BioTECH, China) and stained with 0. 5% crystal violet solution (KGA229, KeyGEN BioTECH, China) for 30 min. Migrated cells were imaged under an inverted microscope (Zeiss, Germany) at ×200 magnification in five randomly chosen fields and analyzed using ImageJ software.

### Antibodies and Reagents

In this study, the following reagents were utilized: Indole-3-carbinol (HY-N0170, MedChemExpress, USA), MG132 (HY-13259, Beyotime, China), Chloroquine (C843545, Macklin, USA), Rapamycin (HY-10219, MedChemExpress, USA), and cycloheximide (HY-12320, MedChemExpress, USA). For CHX chase assays, the indicated time points were used consistently across the relevant protein stability experiments and were sufficient to capture detectable protein decay in the tested cell lines.

Specific details regarding the antibodies utilized in this research can be found in Supplementary Table S1.

### Bioinformatic analysis

We obtained the transcriptome and clinical data of LUAD from The Cancer Genome Atlas (TCGA, https://portal.gdc.cancer.gov/) database and acquired the GSE30219, GSE31210, and GSE72094 datasets from the Gene Expression Omnibus (GEO, https://www.ncbi.nlm.nih.gov/geo/) [32–34]. R 4.4.1 was used for all analyses. The activity of mTORC1 was evaluated through ssGSEA/GSVA in the *GSVA* package with a gene set from MSigDB, and then categorized into high/low groups. Differentially expressed genes (DEGs) analysis in TCGA were performed using DESeq2. GSEA (*fgsea* package) was used for pathway enrichment analysis, with gene sets from Kyoto Encyclopedia of Genes and Genomes (KEGG, https://www.kegg.jp/).

E2F8 expression across clinical features was depicted utilizing *ggplot2*. Enrichment analysis of the differentially expressed genes in the KEGG database employing the *clusterprofiler* tool [35]. Kaplan–Meier curves were generated for survival analysis, and the log-rank test was applied utilizing the survival packages. The Cox proportional hazards model was used to estimate the hazard ratio (HR) and the 95% confidence interval (CI). Clinical annotations were compiled from the TCGA-LUAD and GEO cohorts (GSE30219, GSE31210, GSE72094). Pathological stage was the primary consideration, with missing values excluded and stages standardized into T1-T4, N0-N3, and M0/M1. Following the normalization of the expression matrix within each cohort, log₂ (TPM + 1) (or the normalized platform-provided values) were utilized, and E2F8 expression was extracted for statistical comparisons. All analyses were independently conducted within each cohort, with visualization accomplished through violin plots created with *ggplot2*.

### Single-cell sequencing data analysis

Surgical tissues were procured from the Department of Thoracic Surgery, Jiangsu Province Hospital and the First Affiliated Hospital of Nanjing Medical University with informed consent and ethics approval. Fresh specimens were immediately placed in prechilled PBS containing 0. 04% BSA and processed within 1 h. Tissues were digested with collagenase type I/IV (1–2 mg/mL) and DNase I (0. 1 mg/mL) at 37 °C for 30–60 min, passed through a 40-µm cell strainer, and, when required, erythrocytes were lysed with RBC lysis buffer. Cell viability exceeded 80%. Single-cell suspensions (700–1200 cells/µL) were loaded onto the 10x Genomics Chromium Next GEM Single Cell 3’v3. 1 platform and sequenced on an Illumina NovaSeq 6000 using the recommended read lengths.

Sequencing of the raw data was processed with Cell Ranger (10x Genomics) to generate a UMI count matrix. Further quality control and downstream analysis were processed in *Seurat* in R. UMI count matrices were converted into Seurat objects and iterative clustering was performed using *FindClusters*, with visualization using t-SNE/UMAP. Similarly, Seurat’s *AddModuleScore* calculated per cell mTORC1 module scores with the use of MSigDB Hallmark gene set HALLMARK_MTORC1_SIGNALING. Cells (possibly restricted to epithelial clusters) were divided into mTORC1-high and mTORC1-low. For pathway-level validation, GSEA was performed via fgsea on genome-wide pre-ranked genes extracted from the high-vs-low comparison and the top-scoring results were listed according to NES and FDR. Comparison of differential gene expression between cells originating from different sources was performed with FindAllMarkers, and enrichment analysis for Differentially Expressed Genes (DEGs) with *clusterprofiler* (v4. 6. 2).

### Immunoprecipitation (IP) and immunoblotting (IB) assays

For IB analysis, cells were lysed on ice for 20 min in RIPA buffer (#89001, Thermo Fisher Scientific, USA) supplemented with 1 × Halt protease and phosphatase inhibitor cocktail (#78441, Thermo Fisher Scientific, USA). After that, the proteins were separated by SDS-PAGE and transferred onto PVDF membranes. After that, the membranes were blocked using 5% non-fat milk, followed by overnight incubation at 4 °C with specific primary antibodies. After three washes with TBST, the membranes were exposed to enzyme-conjugated secondary antibody and visualized by chemiluminescence (#34580, Thermo Fisher Scientific, USA).

For IP analysis, cells were lysed on ice for 30 min using IP lysis buffer (#87787, Thermo Fisher Scientific, USA) supplemented with 1X Halt protease and phosphatase inhibitors (#87787, Thermo Fisher Scientific, USA). After centrifugation at 12,000 rpm for 5 min at 4 °C, the supernatant was collected. The supernatant was incubated with the corresponding antibody overnight at 4 °C to form antigen-antibody complexes. On the following day, protein A/G magnetic beads (#PB10101, Vazyme, China) were introduced to capture the target protein, which was then eluted for subsequent IB analysis.

### Protein purification and GST pull-down Assays

Express GST or GST-TSC1 (pGEX-4T-1) in *Escherichia coli* (*E. coli*) BL21 (DE3). After lysis and centrifugation, incubate the supernatant with Glutathione Sepharose 4B beads (17-0756-01, Cytiva, Sweden) overnight at 4 °C, and purify GST-TSC1 according to the manufacturer’s instructions. Transiently express Flag-WWP1 and the catalytically inactive mutant C890A (CA) in HEK293T cells, and purify them using anti-Flag magnetic beads (P2115, Beyotime, China). Validate the fusion proteins by SDS-PAGE/Coomassie Brilliant Blue (CBB) staining and store aliquots at −80 °C.

Incubate the purified Flag-WWP1 or CA with Glutathione Sepharose 4B beads preloaded with GST or GST-TSC1 in binding buffer overnight at 4 °C for the GST pull-down assay. Wash beads thrice with binding buffer, then perform IB analysis. Confirm the loading of GST and GST-fusion proteins on beads by CBB staining.

### Analysis of ubiquitination in vivo and in vitro

Denaturing protein immunoprecipitation (d-IP) was used for the in vivo ubiquitination experiment. Cells were exposed to 20 μM MG132 for 6 h, then lysed in SDS denaturing buffer containing SDS, Tris-HCl, glycerol, and β-mercaptoethanol followed by boiling for 10 min. The lysate was then diluted 10–20 fold with a native lysis buffer composed of Triton X-100, Tris-HCl, NaCl, and glycerol, centrifuged and the supernatant of the lysate was subjected to immunoprecipitation subsequent immunoblotting.

For in vitro ubiquitin, Flag-WWP1 or its catalytically inactive mutant CA was incubated with UBE1 (ab269091, Abcam, USA), UBCH5A (ab269096, Abcam, USA), His-ubiquitin and adenosine triphosphate with or without GST-TSC1 WT or GST-TSC1 K662R. The mixture underwent an NTA pull-down experiment using Ni-NTA magnetic beads, and then immunoblotted.

### RNA sequencing (RNA-seq) analysis

RNA was extracted from PC-9 cells infected with sh-NC or sh-E2F8 using TRIzol (15596026, Invitrogen, USA) according to the contractor’s instructions. Following rRNA depletion, mRNA was converted to cDNA for library preparation, and sequencing was performed by the Nanjing Biomedical Platform Alliance (Nanjing, China). The high-quality reads were mapped to the human reference genome GRCh38, and gene expression was standardized as transcripts per million (TPM). *DESeq2* software was employed for differential expression analysis to pinpoint genes that were differentially expressed subsequent to E2F8 knockdown.

### 4D-DIA Proteomics analysis

PC-9 cells (sh-E2F8 and sh-NC) were taken from −80 °C, lysed with appropriate 8 M urea buffer by sonication and incubated on ice for 30 min. After centrifugation at 14,000 × *g* and 4 °C for 30 min, Bradford assay was performed. Dithiothreitol and iodoacetamide were added to equal volumes of protein solution for reduction and alkylation. Urea concentration was diluted to 2 M with 25 mM Tris-HCl, trypsin added 1:100 (w/w) for digestion at 37 °C for 16 h. Digested peptides were desalted and vacuum lyophilized. On the following day, TFA was added to adjust the pH to ≤2, and the digest was centrifuged at 12,000 × *g* for 15 min to remove precipitates. The supernatant was desalted on C18, vacuum-dried, and stored at −20 °C.

Approximately 200 ng of peptides per injection were enriched on a trap column and separated on an analytical column (AURORA UPLC Column, C18 1.6 μm, 250 mm × 75 μm). The analytical column flow rate was set at 300 nL/min. Two mobile phases were employed: Phase A (water containing 0.1% formic acid, FA) and Phase B (acetonitrile, ACN, containing 0.1% FA). A linear gradient was applied as follows: starting from 2% B, ramping to 22% B over 45 min, then increasing to 35% B at 50 min, 80% B at 55 min, and maintaining 80% B from 55 to 60 min (all percentages are v/v). ACN was purchased from Honeywell and used as LC-MS grade. Mass spectrometric data were acquired in 4D-DIA (diaPASEF) mode with an MS1 range of m/z 100–1700 and ion-mobility separation enabled. Raw data were quantified with DIA-NN (v1. 9. 1) either in library-free mode or by first constructing a spectral library from MSFragger-identified MS/MS spectra followed by targeted extraction. Database searching used the UniProt human proteome (UP000005640), with Trypsin/P specificity and up to two missed cleavages; peptide lengths of 7–30 aa; and carbamidomethylation of cysteine as a fixed modification. The FDR at the PSM, peptide, and protein levels was controlled at 1%. Protein quantification matrices were normalized and subjected to appropriate missing-value handling prior to statistical comparisons. Differentially expressed proteins were filtered based on fold change and *p*-value.

### Immunofluorescence (IF) staining and confocal microscopy

Cells were inoculated onto coverslips and allowed to adhere before addition of assay reagents. The cells were then fixed with 4% paraformaldehyde for 30 min and permeabilized by treatment with 0. 5% Triton X-100 for a further 30 min. They were then blocked with 1% BSA for 2 h at room temperature and incubated in the presence of specific primary antibody overnight at 4 °C. After washing with PBS, they were treated with fluorescence-conjugated antibody for 2 h at room temperature in the dark. DAPI was utilized to stain the cell nuclei for 30 min. Intracellular colocalization images were captured with a Stellaris STED confocal microscope (Leica, Germany). To obtain the fluorescence intensity line graphs, specific regions of interest (ROIs) were drawn across the cells, and the fluorescence intensity profiles of the respective channels along these lines were quantified using the Plot Profile tool in ImageJ software (NIH, Bethesda, MD, USA).

### Chromatin immunoprecipitation (ChIP) assay

DNA-protein crosslinks in LUAD cells are also induced by 4% PFA. Cells are lysed in SDS buffer and chromatin is sonicated to generate DNA fragments of 200–1000 bp. According to the manufacturer’s instructions of ChIP detection kit (P2080S, Beyotime, China), immunoprecipitation is subsequently performed with ChIP-specific antibodies or IgG. Then, the antigen-antibody complex is bound to magnetic beads and eluted. The purified DNA samples are subsequently quantified using RT-qPCR and visualized via agarose gel electrophoresis. The antibodies can be found in Table S1, and the primers are detailed in Table S2.

### Dual-luciferase reporter assay

Transfect *pGL3-WWP1-Promoter* (WT or P1/P2 mutant) with the internal vehicle Renilla control (*pRL-TK Renilla*), and co-transfect E2F8 overexpression vector or empty control. At 48 h post-transfection, the cells were collected and lysed according to the manufacturer’s instructions of Dual-Luciferase Reporter Gene Assay Kit (RG027, Beyotime, China). Luciferase determination was assessed with the use of a multifunctional microplate reader (Biotek, USA) and normalized to Renilla luciferase as a reference.

### Animal studies

All animal experiments were approved by the Nanjing Medical University Institutional Animal Care and Research Committee (IACUC code: IACUC-2411035-1) and were performed in accordance with the relevant guidelines and regulations. Four-to-six-week-old female BALB/c nude mice were purchased from the Experimental Animal Center of Nanjing Medical University. Stable knockdown or overexpression PC-9 cells for E2F8/WWP1 and the corresponding control (5 × 10^6^ cells per mouse, resuspended with a mixture of PBS and Matrigel at 1:1 ratio, 100 μL) were subcutaneously injected into the left groin of mice. For pharmacological experiments: Mice were randomly assigned to treatment with rapamycin (10 mg/kg, i.p., three times a week) or I3C (20 mg/kg, i.p., three times a week), whereas the blank control group was given NS intraperitoneally of equivalent volumes at the same interval. The long diameter (*L*) and short diameter (*W*) of the tumors were measured with a caliper every 2–3 days, and their volume was calculated by the formula: *V* = 0.5 × *L* × *W*^2^. When the endpoint days was reached ([tumor volume] = 1500 mm^3^ or humane termination signs observed, day 28), mice were killed and samples were taken. The tumors were removed, weighed and photographed, then fixed in 4% PFA for HE and IHC (e.g., Ki-67, TSC1, *p*-mTOR (Ser2448)). Random allocation and blind measurement were implemented throughout the process, and exclusion criteria and humane termination criteria were preset before the experiment.

### Statistical analysis

Data are presented as mean ± standard deviation (SD) from at least three independent experiments. GraphPad Prism 10. 0 (GraphPad Software, USA), SPSS 24. 0 (IBM, USA), and R 4. 4. 1 were used for statistical analyses. Two-tailed Student’s t-test was applied to compare two independent groups, whereas paired t-test was used for matched tumor-adjacent samples. For comparisons among more than two groups, one-way ANOVA followed by an appropriate post hoc test was performed. For data that did not meet the normality assumption, the Wilcoxon rank-sum test or Kruskal–Wallis test was used. Correlations between variables were assessed using Spearman’s correlation coefficient. Survival curves were estimated by the Kaplan–Meier method and compared using the log-rank test; hazard ratios (HRs) and 95% confidence intervals (CIs) were calculated from Cox proportional hazards models. A two-sided *P* < 0.05 was considered statistically significant (ns, not significant; *p < 0.05, **p < 0.01, ***p < 0.001).

## Results

### E2F8 links mTORC1 signaling to the E2F program and predicts poor prognosis in LUAD

**Fig. 1:**
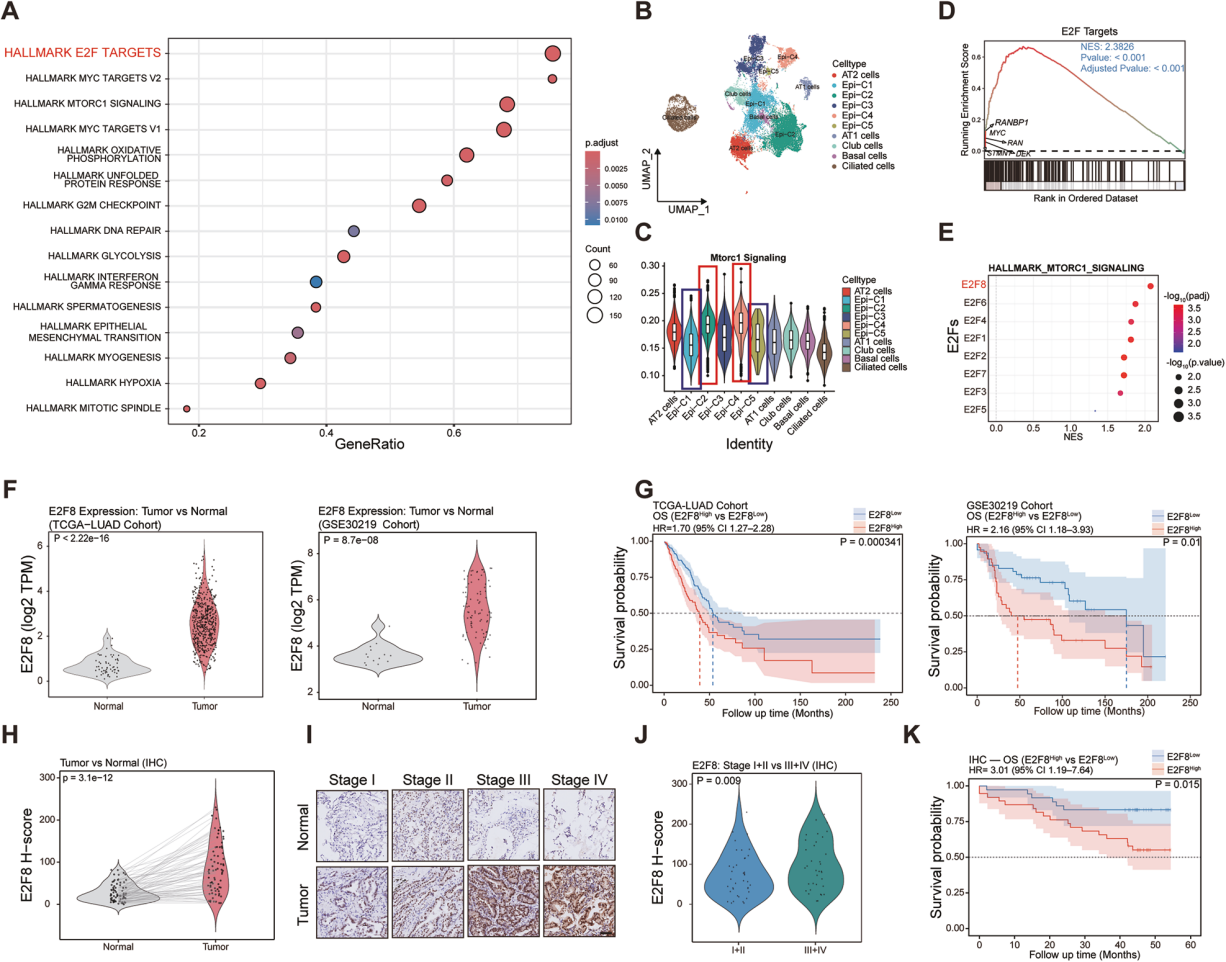
E2F8 links mTORC1 signaling to the E2F program and predicts poor prognosis in LUAD. **A** Pathway enrichment analysis of differentially expressed genes between high vs low mTORC1 ssGSEA scores tumors in TCGA-LUAD. **B**, **C** Single-cell RNA sequencing (scRNA-seq) of LUAD epithelium showing mTORC1 ssGSEA scores across subclusters (AT2 cells, Epi-C1, Epi-C2, Epi-C3, Epi-C4, Epi-C5, AT1 cells, Club cells, Basal cells, and Ciliated cells). Epi-C2 and Epi-C4 represent high-score subclusters, whereas Epi-C1 and Epi-C5 are low-score subclusters. **D** Gene set enrichment analysis (GSEA) demonstrates significant enrichment of E2F_Targets in the mTORC1 high-score subgroup in scRNA-seq data. **E** GSEA of Hallmark mTORC1 for high vs low expression of each E2F (E2F1-E2F8) in TCGA-LUAD. **F** E2F8 expression in tumor vs normal samples in the TCGA-LUAD and GSE30219 cohorts. **G** Survival curves comparing E2F8^high^ and E2F8^low^ groups in GSE30219 and TCGA datasets. **H** E2F8 H-scores in paired tumor and adjacent normal tissues from the TMA cohort were compared using a paired t-test. **I** IHC images showing E2F8 expression in LUAD TMA. Scale bars, 50 μm. **J** Comparison of E2F8 H-scores between paired tumor and adjacent normal tissues in early stage and later-stage LUAD from the TMA cohort, analyzed by paired t-test within each group. **K** Survival analysis comparing E2F8^high^ and E2F8^low^ groups in TMA.

To define its key mechanism in lung adenocarcinoma (LUAD), we computed mTORC1 scores in TCGA-LUAD and stratified tumors into high/low groups. Differential-expression enrichment highlighted E2F_TARGETS (Fig. 1A). When tumors were stratified by high versus low expression of each E2F (E2F1-E2F8) and Gene Set Enrichment Analysis (GSEA) was performed for HALLMARK_MTORC1_SIGNALING, the most significant enrichment was observed for the E2F8-based split (Fig. 1E). In single-cell LUAD data from our institution, the epithelial subpopulation with high mTORC1 scores also showed enrichment of E2F_TARGETS (Figs. 1B, C and S1A, B). Compared with normal epithelium, tumor epithelial cells exhibited higher E2F8, and E2F8^high^ tumor epithelium harbored stronger mTORC1 signaling. (Figs. S1K–M, S1O, S1P). These findings suggest that E2F8 links mTORC1 signaling to the E2F_TARGETS program. Across TCGA/GEO datasets, E2F8 is upregulated in tumors and high-stage cases, and associates with poor prognosis (Figs. 1F, G and S1C–J). Patient tissue microarray (TMA) IHC analyses demonstrated significantly higher E2F8 protein in LUAD versus adjacent non-tumor tissue, consistent pattern preserved in early (I-II) and later (III–IV) stages (Fig. 1H–J). High E2F8 expression was associated with significantly poorer overall survival in patients compared to low expression (log-rank *P* = 0.015; HR = 3.01, 95% CI 1.17–7.64) (Fig. 1K). In cell lines, E2F8 protein and mRNA levels were higher in multiple LUAD lines than in the normal lung epithelial line BEAS-2B and HBE (Fig. S1N).

### E2F8 promotes LUAD progression in vitro and in vivo

**Fig. 2:**
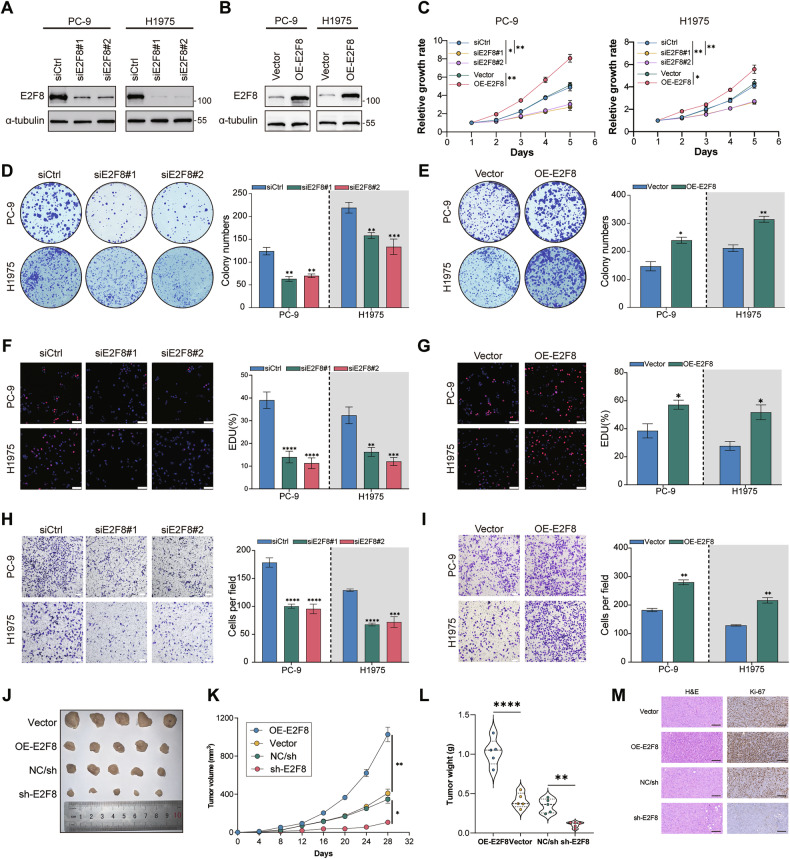
E2F8 promotes LUAD progression in vitro and in vivo. **A**, **B** WB assay of E2F8 expression in H1975 and PC-9 cells transfected with siRNA and overexpression plasmid. **C** The proliferation of H1975 and PC-9 cells, following the specified transfection, was assessed. **D**, **E** Colony formation assessment of H1975 and PC-9 cells transfected as noted. **F**, **G** EdU assay of H1975 and PC-9 cells transfected as noted (scale bars = 100 μm). **H**, **I** Transwell assay of PC-9 and H1975 cells transfected as noted (scale bars = 100 μm). **J** Images of LUAD xenografts with PC-9 cells transfected as noted. **K** Monitoring of LUAD xenografts growth every 4 days after injection. **L** Excised tumors were subjected to weight assessment. **M** Images of tumor sections for each group subjected to H&E and Ki-67 staining (scale bars = 50 μm). Data in (**C**–**I**) are presented as mean ± SD from three independent biological replicates. Data in (**K**, **L**) are presented as mean ± SD, with *n* = 5 mice per group. Statistical significance was determined by two-tailed Student’s t-test for two-group comparisons or one-way ANOVA for multiple-group comparisons. *P < 0.05, **P < 0.01, ***P < 0.001, ****P < 0.0001; ns not significant.

To elucidate the oncogenic role of E2F8 in lung adenocarcinoma, we conducted experiments involving plasmid-mediated E2F8 overexpression and siRNA-mediated E2F8 knockdown, assessing transfection efficiency through WB analysis (Fig. 2A, B). Subsequently, we profiled proliferative responses to E2F8 via CCK-8, EdU, and colony formation assays. The results indicate that E2F8 knockdown inhibits lung adenocarcinoma cell proliferation, while E2F8 overexpression further promotes this effect (Fig. 2C–G). Transwell assays revealed that E2F8 knockdown inhibited cell migration, whereas E2F8 overexpression facilitated cell migration (Fig. 2H, I). Additionally, we established stable PC-9 cell lines with E2F8 knockdown and overexpression (sh-E2F8, OE-E2F8) using lentiviral vectors. BALB/C nude mouse xenograft tumor models were created with PC-9 cells that stably knocked down or overexpressed E2F8. The findings demonstrated that the volume, weight, and Ki-67 positive rate of subcutaneous xenografts were significantly reduced in the sh-E2F8 group, while the opposite was observed in the OE-E2F8 group (Fig. 2J–M). Taken together, the findings implicate elevated E2F8 expression can effectively enhance LUAD progression in vitro and in vivo.

### E2F8 Promotes LUAD progression via activation of the mTORC1 signaling pathway

**Fig. 3:**
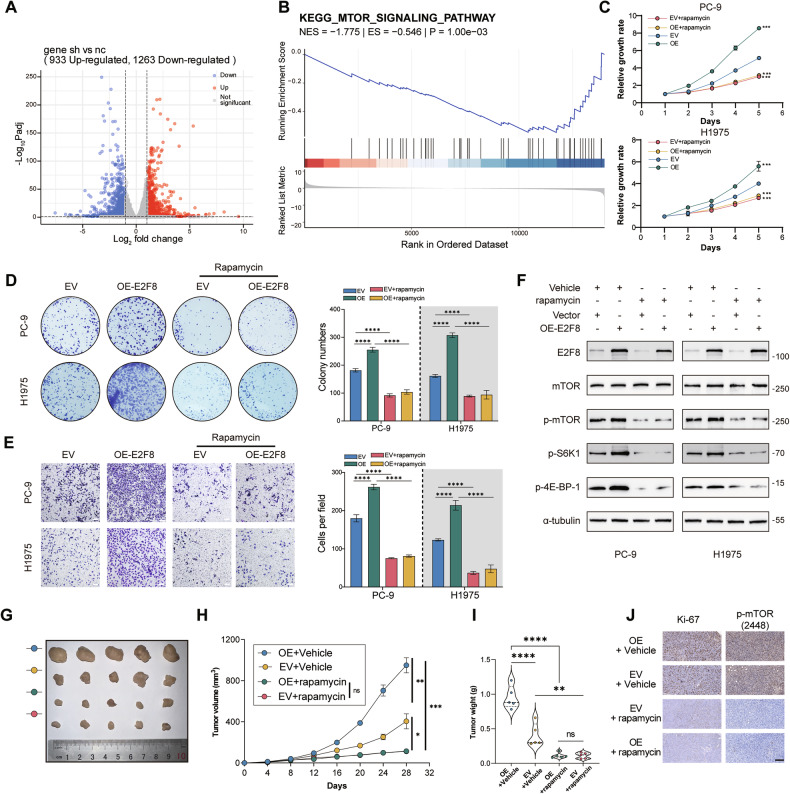
E2F8 fosters LUAD progression via activation of the mTORC1 signaling pathway. **A** The volcano plot shows genes differentially expressed in PC-9 cells after E2F8 knockdown (*n* = 3) compared to normal controls (*n* = 3). **B** GSEA of differentially expressed genes in PC-9 cells after E2F8 knockdown compared with normal. **C** The proliferation of H1975 and PC-9 cells, following the specified transfection, was assessed. EV, empty vector; OE-E2F8, overexpression E2F8. **D** Colony formation assessment of H1975 and PC-9 cells, transfected and treated as noted. **E** Transwell assay of PC-9 and H1975 cells, transfected and treated as noted (scale bars = 100 μm). **F** WB analysis of mTORC1 pathway in PC-9 and H1975 cells transfected with corresponding plasmids treated with rapamycin. **G** Images of LUAD xenografts with PC-9 cells, transfected and treated as indicated. **H** Monitoring of LUAD xenografts growth every 4 days after injection. **I** Excised tumors were subjected to weight assessment. **J** Images of tumor sections for each group subjected to Ki-67 and *p*-mTOR (Ser2448) staining (scale bars = 50 μm). Data in (**C**–**E**) are presented as mean ± SD from three independent biological replicates. Data in (**H**, I) are presented as mean ± SD, with *n* = 5 mice per group. Statistical significance was determined by two-tailed Student’s t-test for two-group comparisons or one-way ANOVA for multiple-group comparisons. *P < 0.05, **P < 0.01, ***P < 0.001, ****P < 0.0001; ns not significant.

To further elucidate the carcinogenic potential of E2F8, transcriptome sequencing was conducted on LUAD cells with E2F8 knockdown (Figs. 3A and S2A, B). GSEA and KEGG revealed negative enrichment of the mTOR and mTORC1 signaling pathways in these LUAD cells (Figs. 3B and S2D). Analysis of both TCGA and self-determined single-cell data indicated that the E2F8^high^ subgroup exhibited significant positive enrichment of mTORC1 signaling (Figs. S2E and S1P). Consequently, E2F8 knockdown appears to inhibit mTORC1 pathway activity, suggesting that E2F8 may function as an upstream positive regulator of this pathway. mTOR possesses seven recognized phosphorylation sites, with Ser2448 phosphorylation serving as a critical marker for mTORC1 activation [36]. To directly assess changes in pathway activity, we evaluated mTORC1-related signaling pathways, including *p*-S6K, *p*-4E-BP1, and mTOR phosphorylation (Ser2448), in E2F8 knockdown LUAD cells using Western blotting (Fig. S2C). These findings indicate that E2F8 serves as a crucial upstream factor that positively regulates mTORC1. Furthermore, to ascertain whether the tumor-promoting effect of E2F8 is contingent upon mTORC1 activation, we employed rapamycin, a well-established mTORC1 inhibitor. The tumor-promoting effect of E2F8 was significantly diminished, as confirmed by CCK8, colony formation, and Transwell assays, along with WB detection of mTORC1 pathway activity (Fig. 3C–E). In vivo, nude mouse transplantation tumor experiments and immunohistochemical analysis of relevant markers revealed that E2F8 overexpression markedly elevated the phosphorylation of mTOR Ser2448 in transplanted tumors. Conversely, treatment with rapamycin not only suppressed this phosphorylation but also decreased the expression of Ki-67, a key proliferation marker (Fig. 3G–J), thus corroborating these findings at the in vivo level. In conclusion, E2F8 promotes the progression of LUAD in an mTORC1 dependent manner by activating the pathway.

### E2F8 activates mTORC1 by ubiquitinating TSC1

**Fig. 4:**
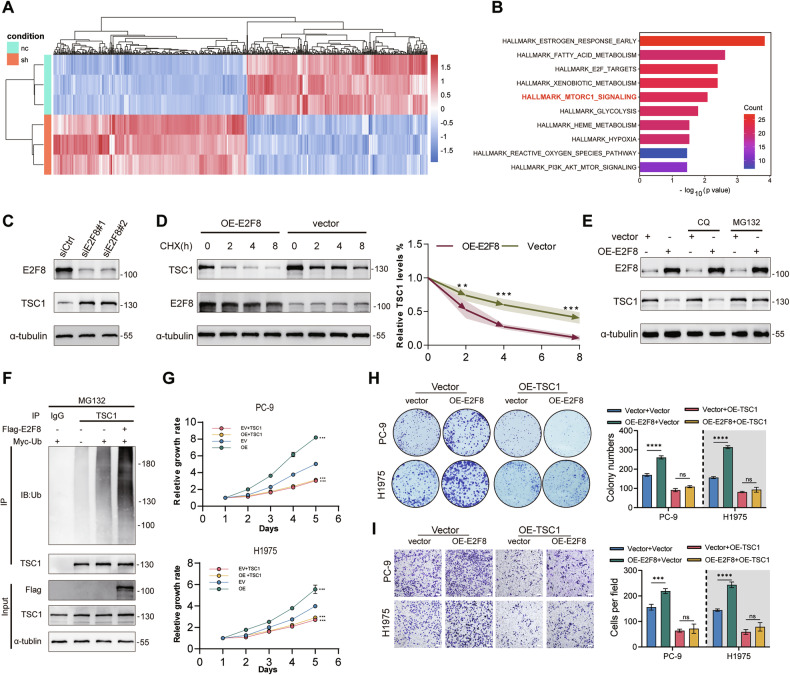
E2F8 activates mTORC1 by ubiquitinating TSC1. **A** Heatmap analysis of differentially expressed proteins (DEPs) between sh-E2F8 and sh-NC LUAD cells. **B** Enriched HALLMARK analysis showed (top 10 categories) based on DEPs. **C** WB analysis of TSC1 protein levels in PC-9 cells transfected as indicated. **D** PC-9 cells transfected with E2F8 or an empty vector control were exposed to CHX for the specified durations. WB was used to detect TSC1 and E2F8 protein levels, and TSC1 levels were normalized to α-tubulin. **E** PC-9 cells were transfected with either E2F8 or an empty vector control, followed by the specified treatments. Protein levels of TSC1 and E2F8 were then measured by WB. **F** Ubiquitination assays of endogenous TSC1 in the lysates from PC-9 cells transfected with Flag-E2F8. **G** The proliferation of H1975 and PC-9 cells, following the specified transfection, was assessed. **H** Colony formation assessment of H1975 and PC-9 cells transfected as noted. **I** Transwell assay of PC-9 and H1975 cells transfected as noted (scale bars = 100 μm). Data are presented as mean ± SD from three independent biological replicates. Statistical significance was determined by one-way ANOVA for multiple-group comparisons. *P < 0.05, **P < 0.01, ***P < 0.001, ****P < 0.0001; ns not significant.

We previously conducted transcriptomic analyses on LUAD cells with E2F8 knockdown. KEGG enrichment profiling of DEGs revealed significant enrichment of the ubiquitin-proteasome pathway alongside the mTOR signaling pathway (Fig. S4A). Given that proteins are the primary mediators of biological activities and that ubiquitination serves as a crucial post-translational modification, functional proteins involved in pathway regulation cannot be fully elucidated through transcriptomic data alone. Accordingly, to elucidate the direct regulatory mechanisms by which E2F8 controls mTORC1 signaling, we undertook proteomic analyses of LUAD cells following E2F8 knockdown to identify core functional mediators (Fig. 4A). After obtaining differentially expressed proteins (DEPs) using 4D-DIA proteomics technology, we conducted bioinformatics enrichment analyses, including HALLMARK, KEGG, and GO. DEPs enrichment mapped significantly to mTORC1 signaling and to downstream associated pathways (Figs. 4B and S3A, B). We centered our analysis on TSC1 (tuberous sclerosis complex 1), an essential inhibitory modulator of mTORC1 [37], which was most significantly increased upon E2F8 silencing in LUAD cells (Fig. S3C).

As a transcription factor, E2F8 regulates target genes at the transcriptional level. Initially, transcriptome data and RT-qPCR analyses of E2F8 knockdown LUAD cells revealed no significant change in *TSC1* mRNA expression (Fig. S3G). However, WB results indicated that E2F8 knockdown significantly enhanced TSC1 protein levels (Figs. 4C and S3D). We hypothesized that E2F8 regulates degradation of TSC1. The main degradation pathways of protein are autophagy-lysosomal and ubiquitin-proteasome pathways [38]. We treated LUAD cells with MG132 inhibitor of the ubiquitin-proteasome pathway and CQ inhibitor of the autophagy-lysosomal pathway. We found that MG132 partially reversed E2F8’s regulation of TSC1 protein (Fig. 4D). This suggests that E2F8’s regulation of TSC1 may be dependent on the ubiquitin proteasome pathway. PC-9 and H1975 cells were treated with cycloheximide (CHX), and TSC1 protein was measured at the indicated times to assess stability under E2F8 modulation. Overexpression of E2F8 significantly enhanced degradation of TSC1 in the presence of CHX. (Figs. 4E and S3E), thereby confirming that E2F8 diminishes TSC1 protein stability. Furthermore, to investigate whether E2F8 facilitates TSC1 degradation via enhanced ubiquitination, we analyzed TSC1 ubiquitination levels in PC-9 and H1975 cells with and without MG132 treatment. The findings revealed that E2F8 overexpression markedly elevated TSC1 ubiquitination levels, while MG132 treatment further accumulated ubiquitinylated TSC1 (Figs. 4F and S3H). In summary, E2F8 enhanced degradation of TSC1 by promoting its ubiquitination.

To determine whether E2F8 regulates mTORC1 and mediates proliferative and migratory phenotypes in a TSC1-dependent fashion, we implemented a rescue experiment via overexpression of TSC1 in E2F8 overexpressing PC-9 and H1975 cells. The promotion of tumor cell proliferation and migration by E2F8 overexpression can be reversed by TSC1 (Fig. 4G–I). Additionally, analysis of mTORC1 pathway-related proteins revealed that E2F8-mediated activation of mTORC1 is significantly inhibited by TSC1 **(**Fig. S3I, J). These findings suggest that E2F8 activates mTORC1 by ubiquitinating the TSC1 protein, thereby facilitating the progression of LUAD.

### WWP1 directly binds to TSC1 and promotes its degradation

**Fig. 5:**
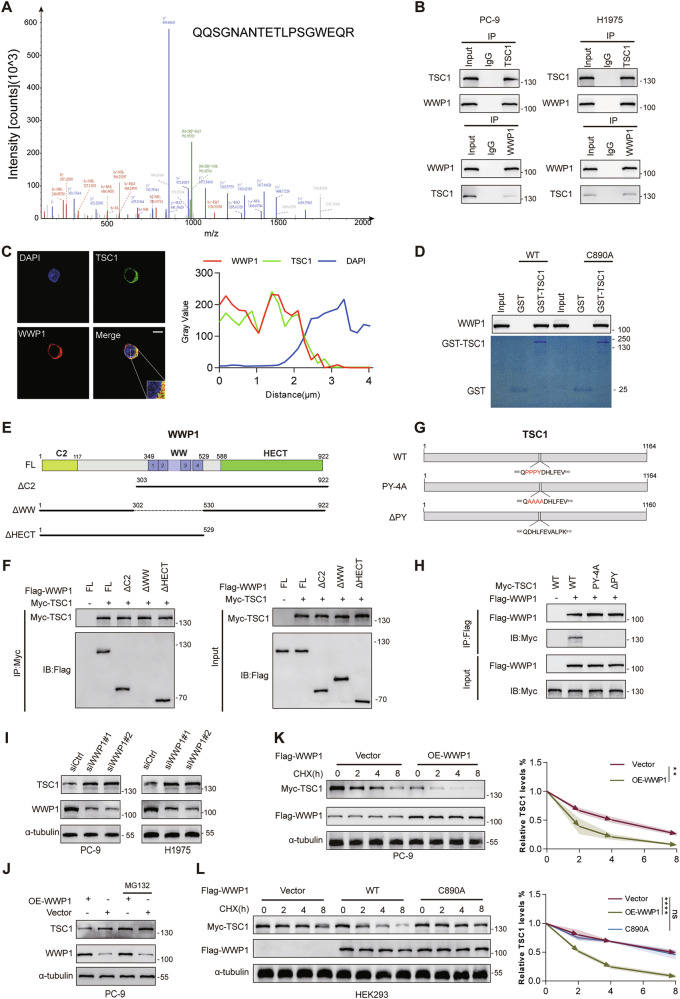
WWP1 directly binds to TSC1 and promotes its degradation. **A** WWP1 peptides identified by MS. **B** Endogenous TSC1-WWP1 interaction was verified by Co-IP. **C** Immunofluorescence (IF) images showing partial co-localization of TSC1 and WWP1, predominantly in the perinuclear region, in HEK293 cells. Enlarged representative views and line-scan fluorescence intensity profiles are shown (scale bars = 10 μm). **D** GST-pulldown test detects interaction between TSC1 and WWP1.Bacteriologically expressed GST control and GST-TSC1 were stained with CBB. Flag-WWP1 WT and catalytically inactive CA were measured by gel electrophoresis. **E** Diagrams showing WWP1 full-length (FL) and three deletion mutant variants. **F** HEK293T cells were co-transfected with Myc-TSC1 and Flag-WWP1 FL or deletion mutants. Cell lysates were immunoprecipitated with indicated antibody followed by IB. **G** Sketch map of the PPxY motif in TSC1 WT and its various mutants [55]. **H** Flag-WWP1 and Myc-TSC1 (WT or different mutants) were transfected into HEK293T cells and cell lysates were immunoprecipitated with indicated antibody followed by IB. **I** WB assays showing the expression of TSC1 and WWP1 in PC-9 cells and H1975 cells transfected with WWP1 siRNA. **J** PC-9 cells were transfected with either E2F8 or an empty vector control, followed by the specified treatments. Protein levels of TSC1 and E2F8 were then measured by WB. **K** PC-9 cells transfected with WWP1 or an empty vector control were exposed to CHX for specified durations. WB was used to detect TSC1 and WWP1 protein levels, and TSC1 levels were normalized to α-tubulin. **L** HEK293 cells transfected with WWP1 (WT) or WWP1 (C890A) mutant were exposed to CHX for specified durations. WB was used to detect TSC1 and WWP1 protein levels, and TSC1 levels were normalized to α-tubulin. Data are presented as mean ± SD from three independent biological replicates. Statistical significance was determined by two-tailed Student’s t-test for two-group comparisons or one-way ANOVA for multiple-group comparisons. *P < 0.05, **P < 0.01, ***P < 0.001, ****P < 0.0001; ns not significant.

E2F8 is a transcription factor and therefore does not directly mediate TSC1 ubiquitination. The KEGG enrichment analysis of the DEGs in E2F8 silenced LUAD cells showed that DEGs were enriched in hsa04120 (Ubiquitin mediated proteolysis) and mTOR signaling pathway (Fig. S4A). Consequently, certain E3 ubiquitin ligases may serve as downstream target genes of E2F8 and directly regulate TSC1 ubiquitination levels. To find candidate E3 ligase, we subjected TSC1 to mass spectrometry (MS) analysis to identify its interacting proteins. By combining the MS data, E2F8 knockdown DEGs and KEGG hsa04120 intersections we identified WWP1 as a preferential candidate for TSC1 interaction as an E3 ligase (Figs. 5A and S4B). We then validated the combination of WWP1 with TSC1 in LUAD cells by co-immunoprecipitation (Co-IP) assay (Fig. 5B). Confocal imaging demonstrated partial co-localization of WWP1 and TSC1, predominantly in the perinuclear region, in HEK293, PC-9, and H1975 cells (Figs. 5C and S4C). As well, using GST pull-down, we confirmed direct association of WWP1 with TSC1, which was unaffected by the catalytic activity of WWP1. (Fig. 5D). To delineate the structural basis of the WWP1-TSC1 interaction, we generated WWP1 deletion mutants (ΔCC, ΔWW, ΔHECT) and observed that ΔCC and ΔHECT retained binding whereas ΔWW did not, indicating that the WW domain is essential for the binding between WWP1 and TSC1 (Fig. 5E, F). WWP1, an archetypal member of the NEDD4 family of proteins that bind PPxY motifs through their WW domain, was first established to recognize PPxY of atrophin-1 via this domain [39]. Significantly, we found a PPxY motif (601-PPPY-604) in TSC1 protein. To investigate the interaction between these two proteins via this motif, we further constructed alanine missense mutations (PY-4A) or deletions of the PPPY motif (ΔPPPY). Additionally, immunoprecipitation experiments showed that TSC1 interacts with WWP1 through its PY motif (Fig. 5G, H), indicating that the WW domain in WWP1 and the PY motif in TSC1 are crucial for their binding.

WWP1 is a classical E3 ubiquitin ligase involved in multiple biological processes through ubiquitin-proteasome–mediated degradation of specific substrates [26]. WWP1 knockdown increased TSC1 protein level (Figs. 5I and S4D), whereas WWP1 overexpression reduced TSC1 and MG132 restored this reduction (Figs. 5J and S4E). Significantly, neither WWP1 knockdown nor WWP1 overexpression altered *TSC1* mRNA levels (Figs. S4D, F), suggesting that WWP1 regulates TSC1 expression at posttranscriptional level. To examine the effect of WWP1 on TSC1 protein stability more specifically, we measured the level of TSC1 in CHX-treated LUAD cells. Forced-expression of WWP1 clearly increased degradation of TSC1 protein levels in PC-9 and H1975 cells (Figs. 5K and S4G), while such an effect was not seen with the corresponding WWP1 C890A mutant **(**Fig. 5L). Moreover, both WWP1 wild type (WT) and WWP1 C890A had no effect on *TSC1* mRNA levels (Fig. S4H). In summary, WWP1 binds to the TSC1 PY motif via its WW domain and regulates TSC1 protein stability through the ubiquitin-proteasome pathway.

### WWP1 catalyzes K48-linked ubiquitination of TSC1 at K662

**Fig. 6:**
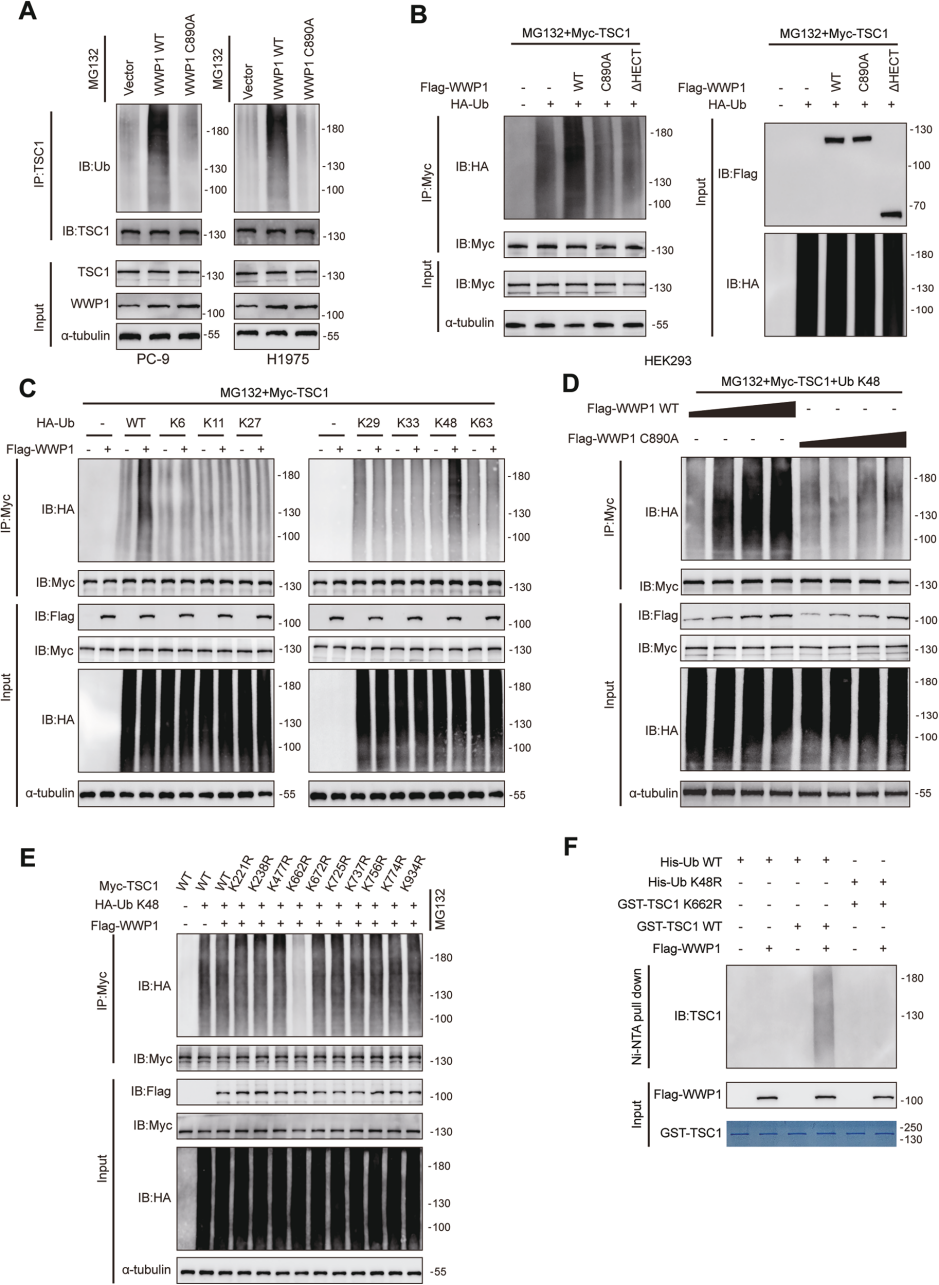
WWP1 catalyzes K48-Linked polyubiquitination of TSC1 at K662 residue. **A** PC-9 and H1975 cells transfected with the empty vector control, WWP1 WT, or WWP1 C890A were exposed to MG132. Denaturing immunoprecipitation (d-IP) was performed using indicated antibody followed by IB. **B** HEK293 cells transfected with Myc-TSC1 and Flag-WWP1(WT, C890A, or ΔHECT) were exposed to MG132. D-IP was performed using indicated antibody followed by IB. **C** HEK293 cells transfected with Myc-TSC1 and Flag-WWP1 together with ubiquitin WT or different ubiquitin mutants were exposed to MG132. D-IP was performed using indicated antibody followed by IB. **D** Co-transfection of HEK293 cells with Myc-TSC1 and rising concentrations of WWP1 WT, or WWP1 C890A was followed by MG132 treatment. D-IP was performed using indicated antibody followed by IB. **E** HEK293 cells transfected with Flag-WWP1, ubiquitin K48, and different Myc-TSC1 mutants were exposed to MG132. D-IP was performed using indicated antibody followed by IB. **F** Purified GST-TSC1 (WT or K662R) was co-incubated with Flag-WWP1 and His-ubiquitin (WT or K48R) proteins. After performing Ni-NTA pull down on the reaction mixtures, the precipitated proteins were analyzed by WB with indicated antibody.

To further clarify the regulatory interaction between WWP1 and TSC1, we investigated the impact of WWP1 on TSC1 ubiquitination. Ectopic expression of WWP1 WT, but not its catalytically inactive C890A mutant, significantly promoted the endogenous TSC1 polyubiquitination in PC-9 and H1975 cells (Fig. 6A). Consistently, Myc-TSC1 was heavily ubiquitinated with WWP1 WT co-expression, but not with WWP1 C890A or WWP1 ΔHECT lacking its HECT domain (the catalytic domain required for E3 ubiquitin ligase activity) (Fig. 6B). With linkage-specific ubiquitin mutants, co-transfection experiments indicated that WWP1 catalyzes K48-linked polyubiquitination of TSC1 (Fig. 6C). This was a WWP1 dose-dependent effect (Fig. 6D). To determine the ubiquitination sites of TSC1 we focused on 10 candidate lysine residues according to sites that were identified in the PhosphoSitePlus (Fig. S5A) [40]. To clarify whether WWP1 mediates polyubiquitination of TSC1 at the mapped sites, we engineered a library of TSC1 lysine-to-arginine mutants and co- transfected each with Flag-WWP1, and showed that the K662R mutation markedly impaired WWP1-mediated K48-linked polyubiquitination of TSC1, whereas the other mutants had little or no effect compared with wild-type controls (Fig. 6E), which was further confirmed by an in vitro ubiquitination assay (Fig. 6F).

To further evaluate the functional importance of the TSC1 K662 site, we performed rescue/reconstitution experiments in TSC1-deficient LUAD cells by ectopically expressing TSC1-WT or TSC1-K662R, followed by E2F8 overexpression or vector control. Immunoblot analysis showed that, compared with TSC1-WT, the TSC1-K662R mutant was more resistant to the reduction in TSC1 protein abundance induced by E2F8 (Fig. S5B). Consistently, CCK-8 assays demonstrated that the K662R mutant more effectively attenuated the pro-growth effect of E2F8 overexpression than the WT construct (Fig. S5C). These findings support K662 as a functionally important site contributing to E2F8-WWP1-mediated TSC1 destabilization and downstream oncogenic phenotypes.

### E2F8 predominantly regulates TSC1 protein stability by transcriptionally activating WWP1

**Fig. 7:**
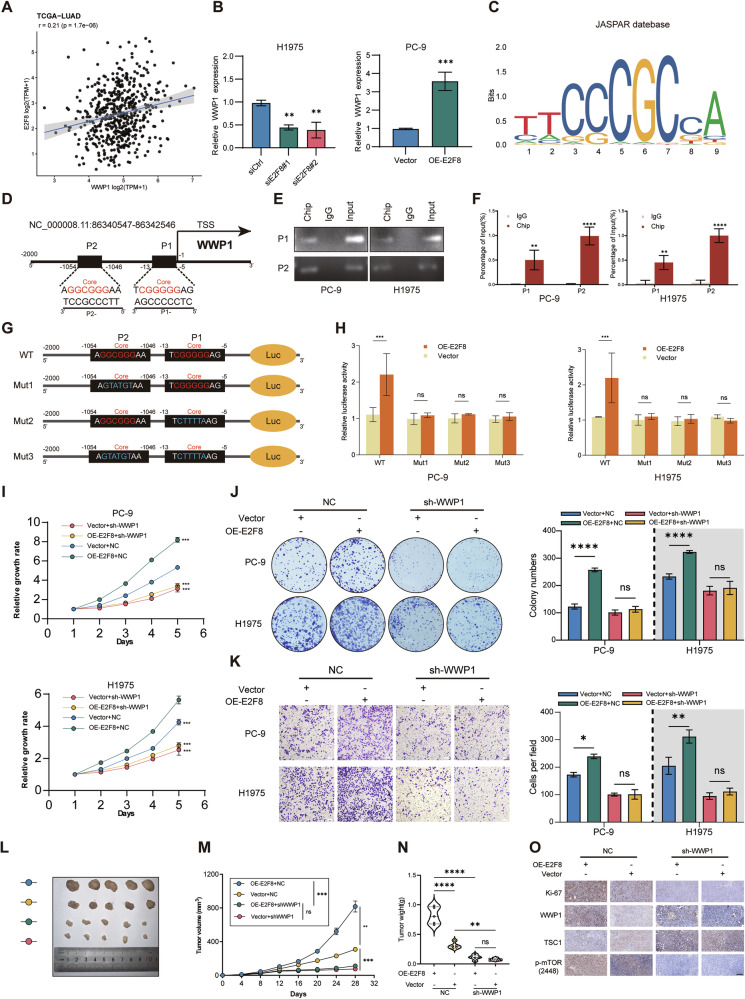
E2F8 regulates TSC1 protein stability by transcriptionally activating WWP1. **A** *E2F8* expression showed a positive correlation with *WWP1* RNA levels in TCGA-LUAD cohort. **B** *WWP1* was examined by RT-qPCR in PC-9 and H1975 cells transfected as indicated. **C**, **D** Predicted E2F8 binding sequences and sites in the WWP1 promoter according to JASPAR. **E** Agarose gel electrophoresis and **F** RT-qPCR analysis of ChIP assay products in LUAD cells. **G** The schematic of the WWP1 promoter with luciferase reporter vectors. **H** The Dual-luciferase assay was conducted to assess the relative luciferase activity of reporter plasmids containing the WWP1 promoter and its mutant type in LUAD cells overexpressing E2F8. **I** The proliferation of H1975 and PC-9 cells, following the specified transfection, was assessed. **J** Colony formation assessment of H1975 and PC-9 cells transfected as noted. **K** Transwell assay of PC-9 and H1975 cells transfected as noted (scale bars = 100 μm). **L** Images of LUAD xenografts with PC-9 cells transfected as noted. **M** Monitoring of LUAD xenografts growth every 4 days after injection. **N** Excised tumors were subjected to weight assessment. **O** Images of tumor sections for each group subjected to Ki-67, WWP1, TSC1 and *p*-mTOR (Ser2448) staining are shown (scale bar, 50 μm). Data in (**B**, **F**, **H**, **I**–**K**) are presented as mean ± SD from three independent biological replicates. Data in (**M**, **N**) are presented as mean ± SD, with *n* = 5 mice per group. Statistical significance was determined by two-tailed Student’s t-test for two-group comparisons or one-way ANOVA for multiple-group comparisons. *P < 0.05, **P < 0.01, ***P < 0.001, ****P < 0.0001; ns not significant.

E2F8 has been identified as a transcriptional activator with an oncogenic role in certain cancers [25]. We next investigated whether E2F8 transcriptionally regulates WWP1 in LUAD. In our study of the TCGA - LUAD cohort, we observed a linear correlation between *E2F8* and *WWP1* mRNA levels (Fig. 7A), with high WWP1 expression linked to poor prognosis in LUAD (Fig. S6A). Consistently, silencing or overexpressing E2F8 in LUAD cells significantly decreased or increased the mRNA level of *WWP1*, respectively (Figs. 7B and S6B), indicating a positive association between E2F8 and WWP1 expression. To further determine whether WWP1 transcription is preferentially regulated by E2F8 rather than by other E2F family members, and to assess whether this regulatory pattern extends beyond EGFR-mutant LUAD cells, we compared the effects of E2F1, E2F7, and E2F8 in parallel in PC-9 cells and A549 cells. Among the tested factors, E2F8 induced the most marked increase in WWP1 mRNA expression in both A549 and PC-9 cells, whereas E2F1 and E2F7 showed substantially weaker effects (Fig. S6C). Accordingly, dual-luciferase reporter assays demonstrated that E2F8 exerted the strongest stimulatory effect on WWP1 promoter activity in both cell lines (Fig. S6D). The JASPAR database predicted two potential E2F8 candidate binding sites in the WWP1 promoter (Fig. 7C, D). ChIP-qPCR analysis indicated that E2F8 can occupy the WWP1 promoter, thereby confirming the direct recruitment of E2F8 to the WWP1 promoter (Fig. 7E, F). To further confirm the transcriptional regulatory effect of E2F8 on WWP1, promoter reporter plasmids carrying WT or mutant E2F8 binding sites were generated (Fig. 7G). We transfected the plasmids into cells with different E2F8 expression levels, followed by luciferase activity assays. Overexpression of E2F8 markedly elevated WT promoter activity, whereas the mutant promoters were not significantly affected. In addition, both P1 and P2 binding sites proved indispensable for E2F8 engagement (Fig. 7H).

In LUAD cells stably overexpressing E2F8, siRNA-mediated WWP1 knockdown mitigated E2F8-driven malignant phenotypes in vitro (CCK-8, colony formation and Transwell assays) and reduced xenograft growth in vivo (Fig. 7I–N). WWP1 depletion mechanistically restored TSC1 protein abundance and attenuated mTORC1 signaling. To further address whether alternative WWP1 substrates might contribute to these phenotypes, we additionally examined PTEN and LATS1 expression in the corresponding knockdown/overexpression models. Under our experimental conditions, WWP1 depletion consistently restored TSC1 protein abundance and reduced *p*-S6K1 levels, whereas PTEN and LATS1 did not show comparably consistent changes in either A549 or PC-9 cells (Fig. S6E). Moreover, functional rescue assays in both PC-9 and A549 cells showed that the suppressive effects of WWP1 knockdown on E2F8-driven cell growth and colony formation were partially reversed by TSC1 silencing, supporting TSC1 as a major downstream effector of WWP1 in this context (Fig. S6F, G). Both the restoration of TSC1 protein abundance and the decreased phosphorylation of mTOR at Ser2448 were evidenced by IHC detection (Fig. 7O), which aligns with reduced WWP1-mediated degradation of TSC1. Taken together, our data support a model in which E2F8 predominantly upregulates WWP1 transcription, leading mainly to TSC1 destabilization and mTORC1 activation to promote LUAD progression.

### I3C inhibits WWP1 and limits LUAD via TSC1-dependent mTORC1 suppression

**Fig. 8:**
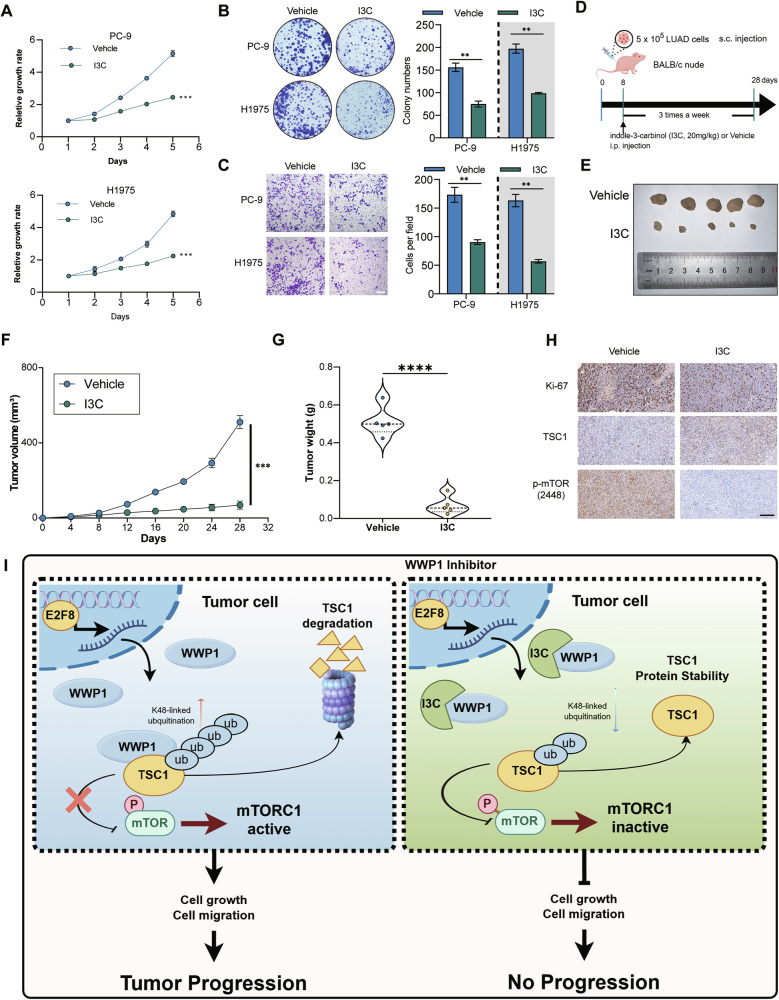
I3C inhibits WWP1 and limits LUAD via TSC1-dependent mTORC1 suppression. **A** The proliferation of H1975 and PC-9 cells, following the specified transfection, was assessed. **B** Colony formation assessment of H1975 and PC-9 cells transfected as noted. **C** Transwell assay of PC-9 and H1975 cells transfected as noted (scale bars = 100 μm). **D** Schematic illustration of the murine subcutaneous tumor model and treatment regimen. By Figdraw.com. **E** Images of LUAD xenografts with PC-9 cells transfected and treated as noted. **F** Monitoring of LUAD xenografts growth every 4 days after injection. **G** Excised tumors were subjected to weight assessment. **H** Images of tumor sections for each group subjected to Ki-67, WWP1, TSC1 and *p*-mTOR (Ser2448) staining are shown (scale bar, 50 μm). **I** A schematic diagram representing the mechanism of this study. By Figdraw.com. Data in (**B**, **C**) are presented as mean ± SD from three independent biological replicates. Data in (**F**, **G**) are presented as mean ± SD, with *n* = 5 mice per group. Statistical significance was determined by two-tailed Student’s t-test for two-group comparisons. *P < 0.05, **P < 0.01, ***P < 0.001, ****P < 0.0001; ns not significant.

Indole-3-carbinol (I3C) specifically inhibits WWP1 via direct binding and suppression of its E3 ubiquitin ligase activity [41]. To evaluate pharmacologic WWP1 inhibition in LUAD, we treated LUAD cells with I3C and observed a marked attenuation of malignant phenotypes (Fig. 8A–C). Subsequently, we evaluated I3C in mice bearing subcutaneous tumors derived from LUAD cells (Fig. 8D). Markedly, I3C inhibited xenograft growth (Fig. 8E–G). As shown by IHC assays, I3C treatment led to decreased Ki-67 and *p*-mTOR (Ser2448) expression, while increasing TSC1 protein levels, consistent with changes in pathway activity (Fig. 8H). Together with our aforementioned finding that E2F8 transcriptionally induces WWP1, leading to TSC1 degradation and subsequent mTORC1 activation, these results indicate that I3C suppresses the E2F8-driven WWP1 pathway, restores endogenous TSC1-mediated repression of mTORC1, and restrains LUAD progression (Fig. 8I).

## Discussion

mTORC1 functions as a central growth-metabolic hub that integrates nutrient, growth factor, and energy/stress signals, and it is frequently dysregulated across cancers, where it contributes to tumor progression and therapy resistance [6, 8, 9, 42]. Although mTORC1 is clearly druggable, the overall clinical benefit of pharmacologic mTORC1 inhibition has been heterogeneous and modest, in part because of the lack of biomarker-based enrichment for pathway-addicted tumors; intrinsic limitations of current inhibitors, which readily trigger vertical/horizontal feedback and network rewiring; and concerns related to toxicity and immune modulation [43]. These issues collectively underscore the need for precise molecular stratification and for combinatorial or mechanism-diversified inhibitory strategies. On the basis of the broad activation of mTOR signaling in LUAD, we analyzed TCGA-LUAD together with our institutional single-cell dataset and found that samples stratified by mTORC1 signaling (HALLMARK_MTORC1_SIGNALING) were significantly enriched for the transcriptional/cell-cycle E2F program (HALLMARK_E2F_TARGETS). When tumors were further grouped by high vs low expression of individual E2F family members (E2F1-E2F8), E2F8-based stratification showed the most pronounced enrichment for mTORC1 signaling. We therefore prioritized E2F8 as a key node that links the E2F program to mTORC1 output in LUAD.

From a translational perspective, our findings provide a mechanistic framework that may help explain why direct mTORC1 inhibition alone often yields limited and heterogeneous benefit in LUAD. In addition to canonical kinase-driven regulation, mTORC1 activity may also be sustained by transcription-coupled proteostatic mechanisms converging on the TSC complex. Here, we show that E2F8 transcriptionally upregulates WWP1, which in turn promotes TSC1 destabilization and sustains mTORC1 signaling. This layered mode of regulation suggests that LUAD cells may preserve mTORC1 activity not only through upstream signaling cascades but also through enhanced degradation of a key inhibitory component. In this context, targeting upstream regulatory nodes such as the E2F8-WWP1 axis may complement direct mTORC1 inhibition. Consistently, pharmacologic inhibition of WWP1 with I3C restored TSC1 levels and attenuated mTORC1 signaling, supporting the feasibility of mechanism-diversified intervention in this pathway.

During cancer progression, canonical upstream pathways such as PI3K-AKT and LKB1-AMPK regulate mTORC1 activity primarily by tuning the TSC complex (TSC1/TSC2) [6, 8, 9, 11]. As the GAP for Rheb, the TSC complex restrains Rheb-GTP accumulation under homeostatic conditions and thereby prevents mTORC1 activation; once this restraining function is compromised, mTORC1 becomes derepressed and persistently activated, driving tumor growth and metabolic reprogramming [44**–**46]. In this context, our transcriptomic and proteomic analyses revealed that E2F8 negatively regulates TSC1 protein abundance, thereby relieving TSC1-mediated inhibition of mTORC1, enhancing downstream pathway activity, and promoting LUAD cell proliferation and migration. This finding further suggests that tumor E2F8 sustains growth signaling by relieving a TSC1-dependent brake. Previous studies have shown that TSC1, beyond forming a complex with TSC2 to inhibit Rheb, can cooperate with PER2 and be recruited to the mTORC1 complex to exert direct inhibitory effects. Thus, E2F8-driven downregulation of TSC1 would be expected not only to attenuate the classical GAP-dependent restraint of the TSC complex, but also to disrupt its direct inhibitory engagement with mTORC1, thereby amplifying mTORC1 output at multiple levels. Notably, mTORC1 activity has been reported to fluctuate dynamically across the cell cycle—peaking in S/G2 and dropping in M/G1—a pattern likely involving TSC complex regulation [47]. This periodicity parallels the physiological upregulation of E2F8 in S/G2, supporting the notion of a dynamic coupling between cell-cycle circuitry and metabolic signaling and providing a physiological rationale for an E2F8-TSC axis.

Another important implication of our findings is that this regulatory circuit may intersect with the genetically heterogeneous upstream landscape of LUAD. In this disease, recurrent alterations involving EGFR, KRAS, and STK11/LKB1 converge on mTORC1 signaling through partially overlapping but non-identical mechanisms. In the present study, the predominant effect of E2F8 on WWP1 transcription and the preferential involvement of the TSC1-mTORC1 axis could also be reproduced in KRAS-mutant A549 cells, suggesting that this pathway is not restricted to EGFR-mutant LUAD models. Although the degree of dependency on this axis may vary across molecular subtypes, these data raise the possibility that the E2F8-WWP1-TSC1 circuit acts as an additional regulatory layer cooperating with canonical oncogenic signaling to sustain mTORC1 activity under distinct genetic backgrounds.

The functionally active TSC complex comprises TSC1 (hamartin), TSC2 (tuberin), and TBC1D7 in an approximate 2:2:1 stoichiometry. As a tumor-suppressive assembly, this complex is essential for restraining mTORC1 and thereby for tumor control [48]. Accumulating evidence indicates that the protein homeostasis of the TSC complex is subject to finely tuned ubiquitination/deubiquitination, and stabilizing this complex has emerged as an attractive strategy to block aberrant mTORC1 activation and inhibit tumor growth [48–51]. However, the post-translational regulation of TSC1 in LUAD has remained poorly defined. In this study, we show that E2F8 transcriptionally activates the E3 ubiquitin ligase WWP1, which in turn governs the ubiquitination status of TSC1. WWP1 engages TSC1 through its characteristic WW domains recognizing the PPxY motif in TSC1, and assembles K48-linked polyubiquitin chains on TSC1 at Lys662, thereby targeting TSC1 for proteasome-dependent degradation. Importantly, our rescue/reconstitution experiments further support K662 as a functionally important site contributing to E2F8-WWP1-mediated TSC1 destabilization and downstream pro-growth effects, although additional ubiquitination sites may also exist. In addition, while WWP1 has been reported to regulate other substrates such as PTEN and LATS1 in different tumor contexts [52], our data in LUAD cells suggest that the predominant functional output of WWP1 in the present model is mediated through the TSC1-mTORC1 axis. Despite elucidating a previously unrecognized mechanism whereby E2F8 activates mTORC1 in LUAD via WWP1-mediated TSC1 degradation, our study has several limitations. First, although the clinical relevance of E2F8, WWP1, and mTORC1 pathway activation was supported by public datasets and our institutional tissue microarray cohort, validation in an independent external LUAD cohort is still lacking. Future studies incorporating larger multicenter cohorts with well-annotated clinicopathological and follow-up data will be needed to further establish the prognostic value of the E2F8-WWP1-TSC1-mTORC1 axis. Second, our in vivo experiments were based on a subcutaneous xenograft model, which does not fully recapitulate the native lung microenvironment. More clinically relevant systems, such as orthotopic models, patient-derived xenografts, or LUAD organoids, will therefore be important for evaluating the biological and therapeutic significance of this pathway [53]. Third, LUAD is molecularly heterogeneous, and distinct oncogenic backgrounds may shape mTORC1 dependency and responsiveness to WWP1-targeted intervention. Finally, direct evidence specifically linking E2F8 to mTORC1 signaling in cancer types beyond LUAD remains limited. Nevertheless, emerging evidence suggests that E2F family members may intersect with PI3K-AKT-mTOR-related signaling in other solid tumors [54]; however, whether the specific E2F8-WWP1-TSC1 mechanism identified here is conserved across different cancer types remains unclear and warrants further investigation. Overall, our findings support the E2F8-WWP1-TSC1 axis as a biologically meaningful regulatory pathway in LUAD, while its broader generalizability and therapeutic tractability will require further validation in multicenter, multi-omics, and diversified functional models.

## Conclusion

In summary, by integrating TCGA-LUAD with our institutional scRNA-seq, we identify E2F8 as a key driver that connects the E2F transcriptional program to mTORC1 signaling in LUAD. Multi-omics and functional assays show that E2F8 is upregulated in tumor epithelium, associates with poor prognosis, and drives proliferation, migration, and xenograft growth in an mTORC1-dependent manner. Mechanistically, E2F8 transcriptionally activates the E3 ligase WWP1, which recognizes the PPxY motif in TSC1 and catalyzes K48-linked polyubiquitination at Lys662, thereby promoting proteasomal degradation of TSC1. Targeting WWP1 via genetic knockdown or pharmacological agent I3C restores TSC1 expression, lowers *p*-mTOR Ser2448 levels, and attenuates malignant phenotypes. Collectively, these findings nominate the E2F8-WWP1-TSC1-mTORC1 axis as a mechanistic link between cell-cycle transcription and growth-metabolic output, and as a tractable avenue for biomarker-guided stratification and therapeutic intervention in LUAD.

## Supplementary information

Supplementary Figures and Tables

Original western blots

## Data Availability

All presented data and code in this study are obtainable from the corresponding author upon reasonable request.
