# Supplementary material for: E2F-mediated activation of mTORC1 through the ubiquitin-proteasome system promotes lung adenocarcinoma progression
Source: Cell Death Dis. 2026 May 19;17(1):635. doi: 10.1038/s41419-026-08863-2 (PMC13358063; doi:10.1038/s41419-026-08863-2)
Supplement: Supplementary file 1 — Supplementary Figures and Tables [file 41419_2026_8863_MOESM1_ESM.pdf]

Supplementary

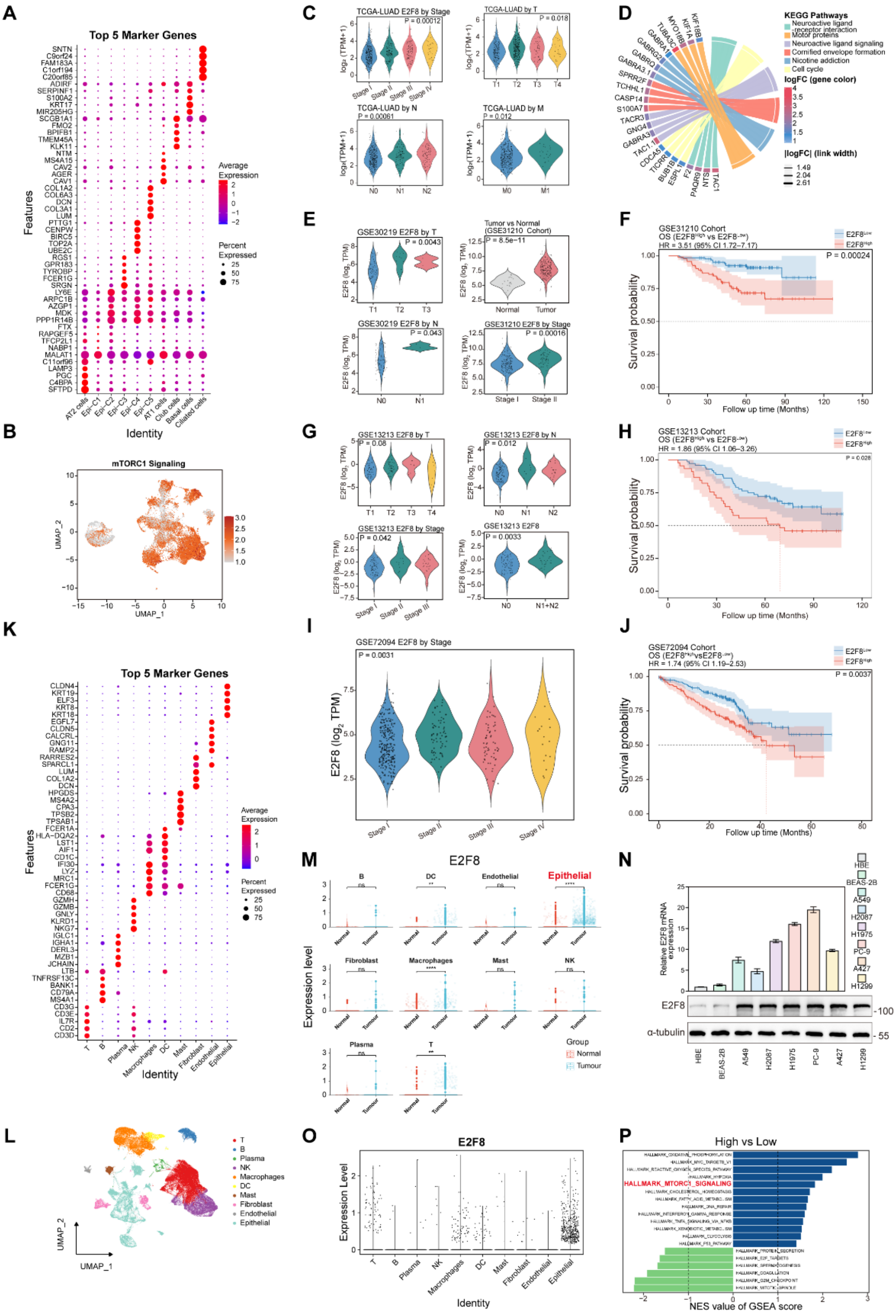

Figure S1. E2F8 links mTORC1 signaling to the E2F program and predicts poor prognosis in LUAD. **(A)** Dot-plot of the top five marker genes across epithelial subclusters in the LUAD scRNA-seq dataset. The size of the dots and the intensity of

the colors represent the percentage of cells expressing each gene and the average expression level, respectively. **(B)** UMAP of epithelial cells from LUAD single-cell RNA-seq, colored by the activity of HALLMARK_MTORC1_SIGNALING pathway quantified via ssGSEA. **(C)** Violin plots of the association between E2F8 expression and clinical subgroups derived from the TCGA-LUAD cohort. Overall differences were assessed by a two-sided Kruskal-Wallis test, with $p < 0.05$ considered statistically significant. **(D)** KEGG enrichment analyses between $E2F8^{high}$ and $E2F8^{low}$ subgroups. **(E) (G) (I)** Violin plots of the association between E2F8 expression and clinical subgroups derived from the GSE30219, GSE31210 cohorts, GSE13213 cohort, and GSE72094. $P < 0.05$ was considered statistically significant. **(F) (H) (J)** Kaplan-Meier survival curves between $E2F8^{high}$ and $E2F8^{low}$ groups in the GSE30219, GSE31210, GSE13213, and GSE72094 cohort. **(K)** Dot plot of the top five marker genes for major cell types in LUAD scRNA-seq. **(L)** UMAP of LUAD scRNA-seq (including tumor/normal) colored by major cell types. **(M)** Violin plots comparing E2F8 expression levels across the indicated cell populations in LUAD scRNA-seq (including tumor/normal). Statistical significance levels were designated as follows: ns, $p > 0.05$, *$p < 0.05$, **$p < 0.01$, ***$p < 0.001$, ****$p < 0.0001$. **(N)** WB and qPCR were employed to determine E2F8 expression in multiple lung cancer cell lines, alongside human normal lung epithelial and normal bronchial epithelial cells. **(O)** Scatter plot depicting the expression levels of E2F8 across various cell types in LUAD tumor single-cell RNA-seq. **(P)** Hallmark GSEA comparing $E2F8^{High}$ versus $E2F8^{Low}$ epithelial cells from LUAD single-cell RNA-seq.

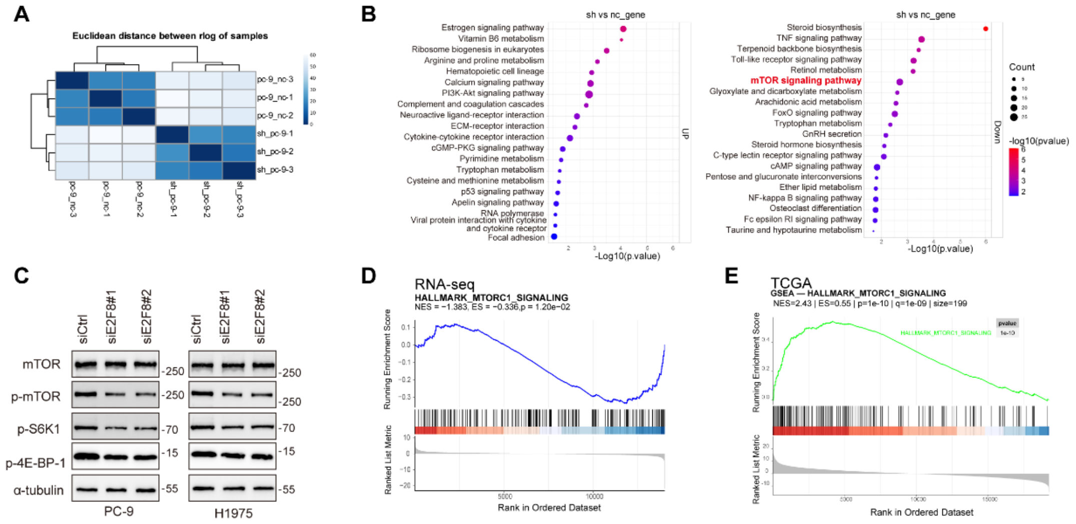

Figure S2. E2F8 fosters LUAD progression via activation of the mTORC1 signaling pathway. **(A)** Heatmap of Euclidean distances between PC-9 cells after E2F8 knockdown (n=3) and normal controls (n=3); distances are color-coded. **(B)** KEGG pathway enrichment analysis of significantly up-regulated and down-regulated genes. **(C)** The proliferation of H1975 and PC-9 cells, following the specified transfection, was assessed. **(D)** WB analysis of mTORC1 pathway in PC-9 and H1975 cells transfected as noted. **(E)** GSEA of differentially expressed genes in PC-9 cells after E2F8 knockdown compared with normal. **(F)** GSEA of differentially expressed genes in samples with high E2F8 expression compared with samples with low E2F8 expression in the TCGA cohort. Data were representative images or were expressed as the mean ± standard deviation.

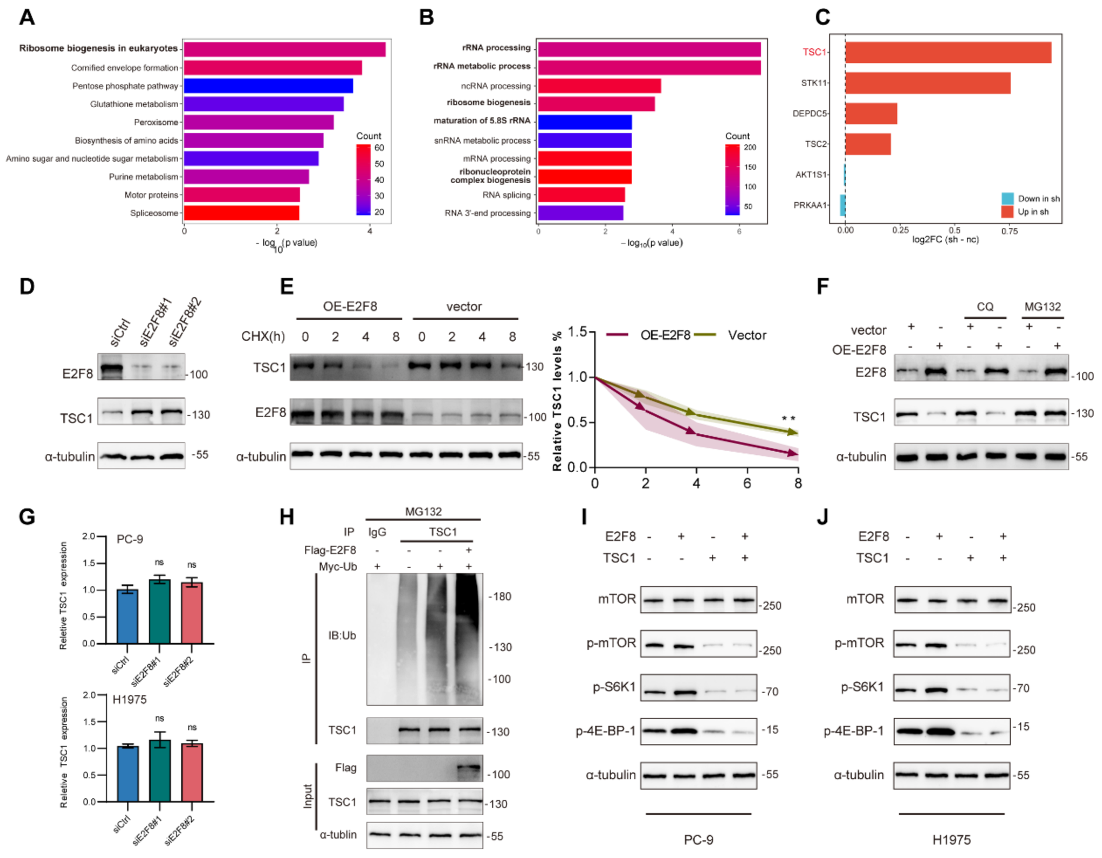

Figure S3. E2F8 activates mTORC1 by ubiquitinating TSC1. **(A)** The top 10 KEGG-enriched categories based on DEPs were demonstrated by enrichment analysis. **(B)** GO enrichment analysis-derived top 10 molecular functions of DEPs are presented in the bar graph. **(C)** The bar graph shows the subset of DEPs associated with mTOR inhibitory proteins in PC-9 cells after E2F8 knockdown. **(D)** WB analysis of TSC1 protein levels in H1975 cells transfected as noted. **(E)** H1975 cells, transfected with E2F8 or an empty vector control were exposed to CHX for the specified durations. WB was used to detect TSC1 and E2F8 protein levels, and TSC1 levels were normalized to α-tubulin. **(F)** H1975 cells were transfected with either E2F8 or an empty vector control, followed by the specified treatments. Protein levels of TSC1 and E2F8 were then measured by WB. **(G)** TSC1 was examined by qPCR in H1975 and PC-9 cells transfected as indicated. **(H)** Ubiquitination assays of endogenous TSC1 in the lysates from H1975 cells transfected with Flag-E2F8. **(I)** WB of

mTORC1 pathway markers in PC-9 cells transfected as indicated. **(J)** WB of mTORC1 pathway markers in H1975 cells transfected as indicated. Data are presented as mean ± SD from three independent biological replicates.. ns: no significant, *$p<0.05$, **$p<0.01$, ***$p<0.001$, ****$p<0.0001$*.

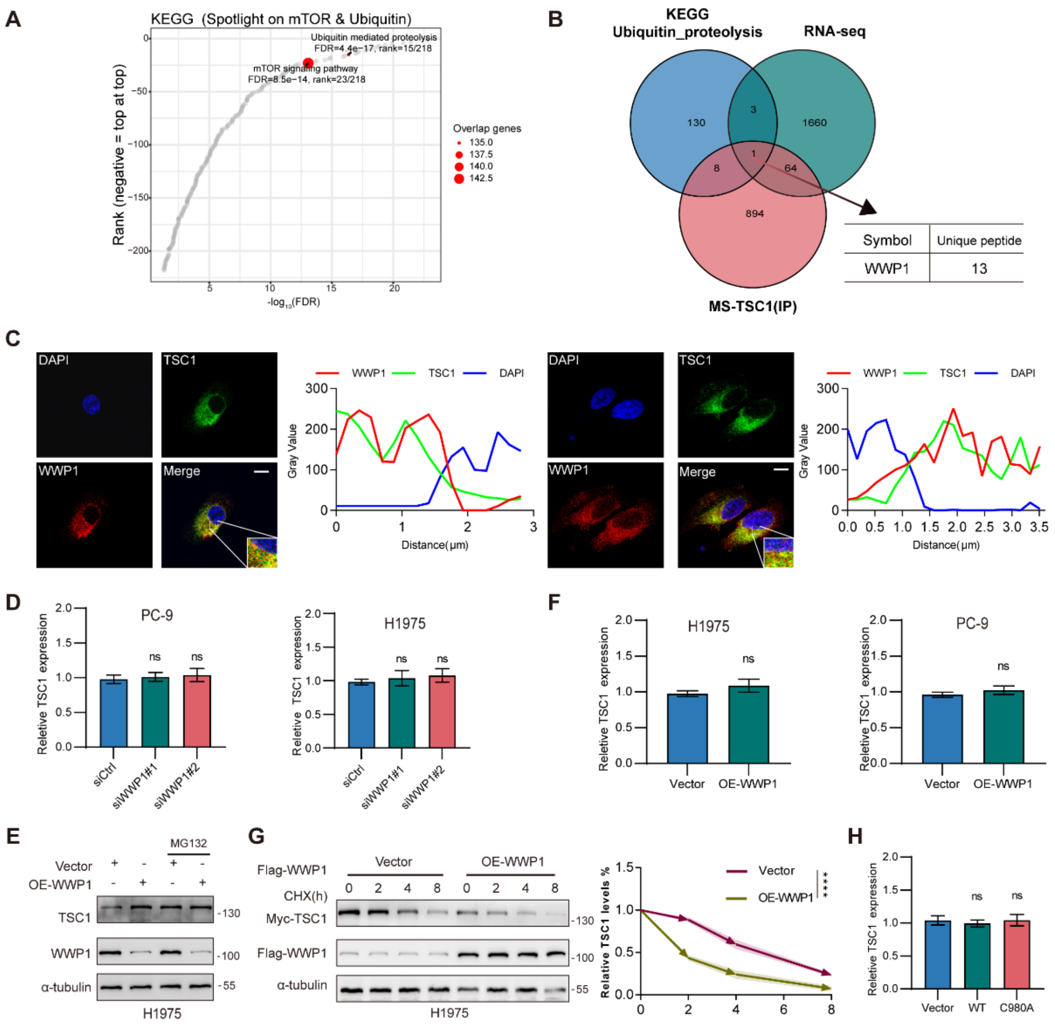

Figure S4. WWP1 directly binds to TSC1 and promotes its degradation. **(A)** KEGG analysis of differentially expressed genes (DEGs) from PC-9 cells with E2F8 knockdown versus control. **(B)** Venn diagram showing the intersection of RNA-seq derived differentially expressed genes, mass spectrometry identified TSC1 interacting proteins, and genes from KEGG pathway hsa04120, used to screen potential E3 ubiquitin ligase candidates. **(C)** Representative IF images showing partial co-

localization of TSC1 and WWP1, predominantly in the perinuclear region, in PC-9 (left) and H1975 (right) cells. Enlarged representative views and line-scan fluorescence intensity profiles are shown (scale bars = 10 μm). **(D)** RT-qPCR assays showing the expression of TSC1 and WWP1 in PC-9 cells and H1975 cells transfected with WWP1 siRNA. **(E)** H1975 cells were transfected with either E2F8 or an empty vector control, followed by the specified treatments. Protein levels of TSC1 and E2F8 were then measured by WB. **(F)** The mRNA levels of *TSC1* were determined by RT-qPCR in H1975 and PC-9 cells transfected as noted. **(G)** H1975 cells transfected with WWP1 or an empty vector control were exposed to CHX for specified durations. WB was used to detect TSC1 and WWP1 protein levels, and TSC1 levels were normalized to α-tubulin. **(H)** The mRNA levels of *TSC1* were determined by RT-qPCR in HEK293 cells transfected as noted. Data are presented as mean ± SD from three independent biological replicates. Statistical significance was determined by two-tailed Student's t-test for two-group comparisons or one-way ANOVA for multiple-group comparisons. *$P < 0.05$, **$P < 0.01$, ***$P < 0.001$, ****$P < 0.0001$; ns, not significant.

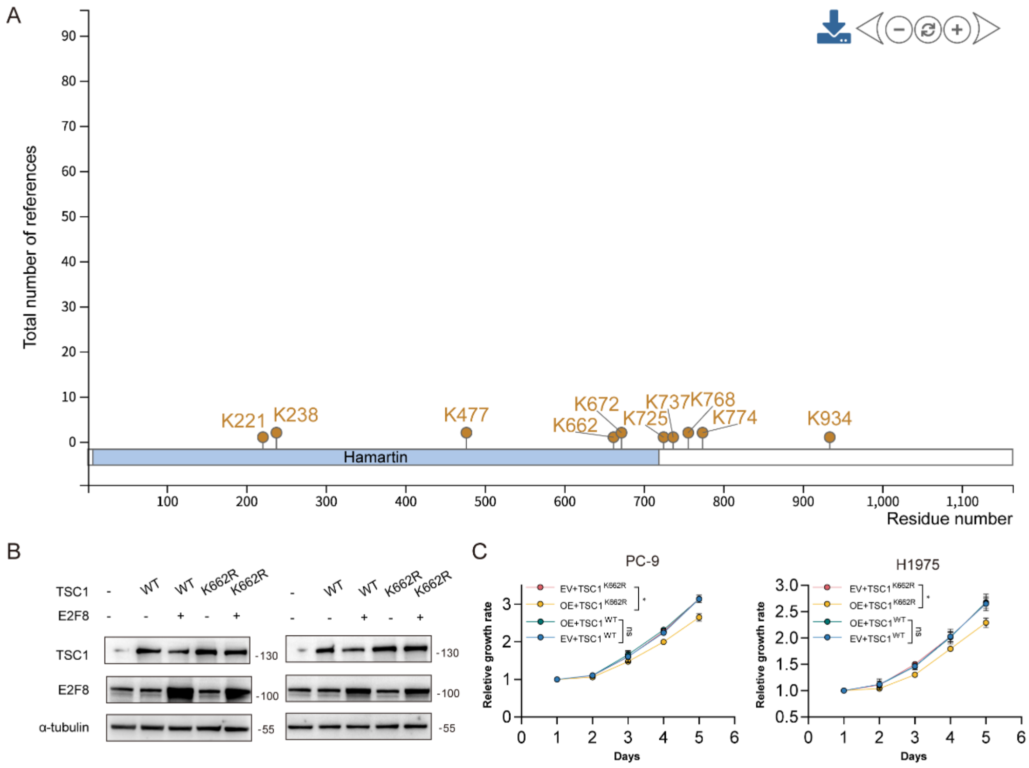

Figure S5. TSC1 ubiquitination sites, WWP1 expression, survival analysis and tumor weight relevant to the E2F8-WWP1-TSC1 axis. **(A)** Ubiquitination sites of TSC1. **(B-C)** In TSC1-deficient PC-9 and H1975 cells, either TSC1-WT or TSC1-K662R was reintroduced, followed by E2F8 overexpression or vector control. TSC1 and E2F8 protein levels were examined by western blotting **(B)**, and cell viability was measured by CCK-8 assay **(C).** Data are presented as mean ± SD from three independent biological replicates. Statistical significance was determined by two-tailed Student's t-test for two-group comparisons or one-way ANOVA for multiple-group comparisons. $*P < 0.05$, $**P < 0.01$, $***P < 0.001$, $****P < 0.0001$; ns, not significant.

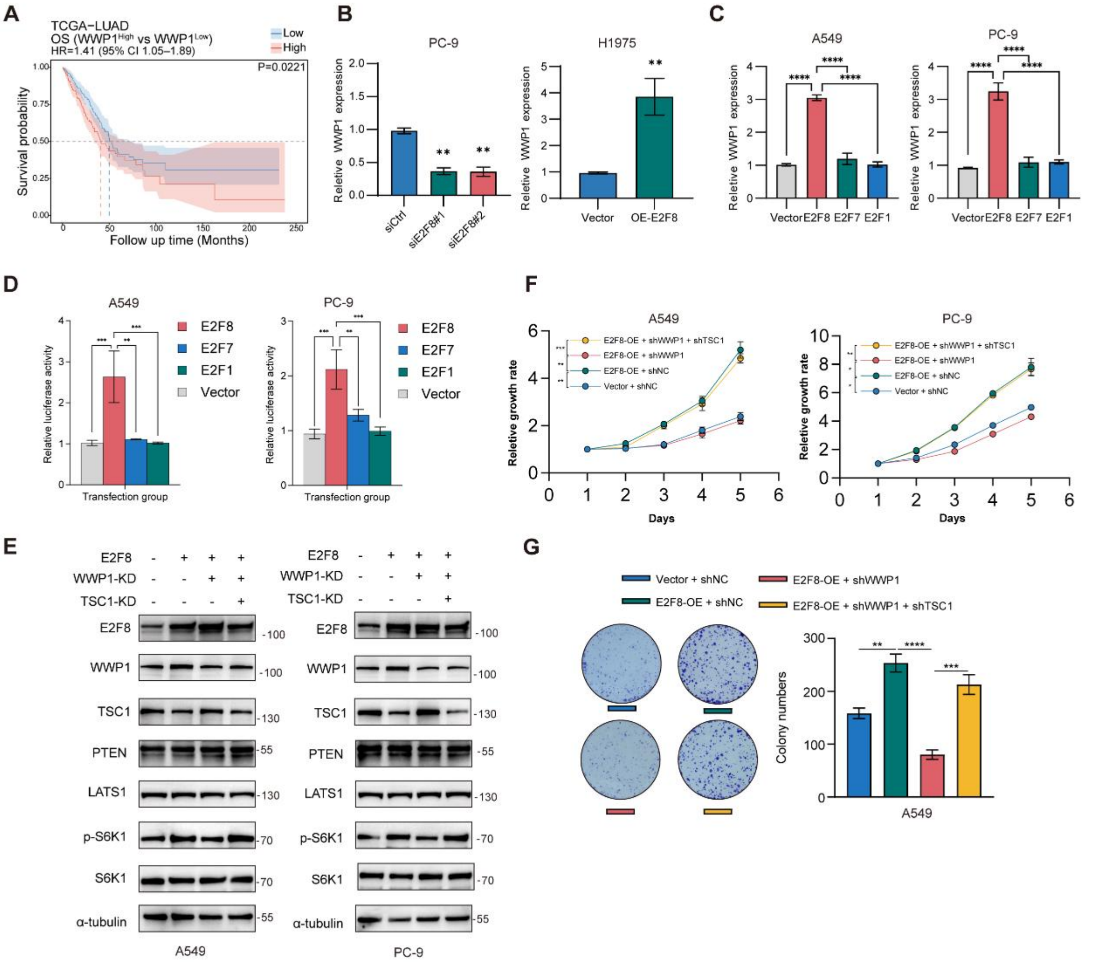

Figure S6. Validation that E2F8 predominantly regulates TSC1 protein stability by transcriptionally activating WWP1. **(A)** Kaplan-Meier survival curves between WWP1^{high} and WWP1^{low} groups in the TCGA-LUAD cohort. **(B)** *WWP1* were determined by RT-qPCR in PC-9 and H1975 cells transfected as noted. **(C)** *WWP1* were determined by RT-qPCR in PC-9 and A549 cells transfected as noted. **(D)** Dual-luciferase reporter assays showing the relative activity of the WWP1 promoter in A549 and PC-9 cells after transfection with Vector, E2F1, E2F7, or E2F8. **(E)** WB of the indicated proteins in A549 and PC-9 cells transfected as indicated. (F) CCK-8 assays in A549 and PC-9 cells transfected as indicated. **(G)** Colony formation assays in A549 cells transfected as indicated. Data are presented as mean ± SD from three independent biological replicates. Statistical significance was determined by two-

tailed Student's t-test for two-group comparisons or one-way ANOVA for multiple-group comparisons. *$P < 0.05$, **$P < 0.01$, ***$P < 0.001$, ****$P < 0.0001$; ns, not significant.

Table S1: The antibodies and their corresponding dilution ratios.

Antibodies	Host	Manufacturer	Catalogue Number	Dilution	Usage
Anti-E2F8 antibody	Rabbit	CST	#34661	1:1000	WB
Anti-Alpha Tubulin antibody	Rabbit	Proteintech	11224-1-AP	1:10000	WB
Anti-TSC1 antibody	Rabbit	Proteintech	29906-1-AP	1:1000	WB
Anti-WWP1 antibody	Rabbit	Abcam	ab104440	1:2000	WB
Anti-mTOR antibody	Rabbit	CST	# 2972	1:1000	WB
Phospho-mTOR (Ser2448) Antibody	Rabbit	CST	#2971	1:1000	WB
Phospho-4E-BP1 (Thr37/46) Antibody	Rabbit	CST	#9459	1:1000	WB

Phospho-p70 S6 Kinase (Thr389) Antibody	Rabbit	CST	#9234	1:1000	WB
HA Tag Recombinant antibody	Mouse	Proteintech	66006-2-Ig	1:10000	WB
DYKDDDDK tag Recombinant antibody	Rabbit	Proteintech	80801-2-RR	1:1000	WB
MYC tag Recombinant antibody	Rabbit	Proteintech	80469-2-RR	1:100000	WB
Anti-TSC1 antibody	Rabbit	Proteintech	29906-1-AP	1:100	IF
Anti-WWP1 antibody	Mouse	Santz cruz	sc-100679	1:50	IF
DYKDDDDK tag antibody	Mouse	Proteintech	66008-4-Ig	1:200	IF
MYC tag antibody	Rabbit	Proteintech	80469-2-RR	1:200	IF
Anti-TSC1 antibody	Rabbit	Proteintech	29906-1-AP	1:200	IP

Anti-WWP1 antibody	Rabbit	Abcam	ab104440	2 µg for 1 mg of total protein lysate	IP
Anti-TSC1 antibody	Rabbit	CST	#6935	1:50	IP
DYKDDDDK tag antibody	Rabbit	Proteintech	80801-2-RR	1 µg for 1 mg of total protein lysate	IP, ChIP
DYKDDDDK tag antibody	Mouse	Proteintech	66008-4-Ig	1 µg for 1 mg of total protein lysate	IP
MYC tag antibody	Rabbit	Proteintech	80469-2-RR	1 µg for 1 mg of total protein lysate	IP
HA Tag antibody	Mouse	Proteintech	66006-2-Ig	1 µg for 1 mg of total protein lysate	IP
Rabbit IgG Control	Rabbit	Proteintech	98136-1-RR	1:50	ChIP
Rabbit IgG Control	Rabbit	Proteintech	98136-1-RR	1 µg for 1 mg of total protein lysate	IP
Rabbit IgG Control	Rabbit	Proteintech	30000-0-AP	1 µg for 1 mg of total protein lysate	IP

Anti-E2F8 antibody	Mouse	Abnova	H00079733-M01	1:1000	IHC
Anti-TSC1 antibody	Rabbit	Proteintech	29906-1-AP	1:300	IHC
Anti-WWP1 antibody	Rabbit	Proteintech	28689-1-AP	1:300	IHC
Phospho-mTOR (Ser2448) Antibody	Rabbit	Abcam	ab109268	1:100	IHC
Anti-Ki-67 Antibody	Rabbit	Proteintech	27309-1-AP	1:5000	IHC
Anti-mouse IgG for IP (HRP)	Mouse	Vazyme	RA1009	1:2000	WB, IP
Anti-rabbit IgG for IP (HRP)	Rabbit	Vazyme	RA1008	1:2000	WB, IP
HRP-conjugated Affinipure Goat Anti-Mouse IgG(H+L)	Mouse	Proteintech	SA00001-1	1:1000	WB
HRP-conjugated Affinipure Goat Anti-Rabbit IgG(H+L)	Rabbit	Proteintech	SA00001-2	1:1000	WB

CoraLite488-conjugated Goat Anti-Rabbit IgG(H+L)	Rabbit	Proteintech	SA00013-2	1:200	IF
CoraLite594-conjugated Goat Anti-Mouse IgG(H+L)	Mouse	Proteintech	SA00013-3	1:200	IF

**Table S2: The primer sequences.**		
Gene	Forward Primer (5'-3')	Reverse Primer (5'-3')
E2F8	CCTGAGATCCGCAACAGAGAT	AGATGTCATTATTCACAGCAGGG
WWP1	TGCTTCACCAAGGTCTGATACT	GCTGTTCCGAACCAGTTCTTTT
TSC1	ACCGAGAGGAATTCTACAACCAG	TTGGCCTTCTTCAGTTCTATCCG
WWP1_P1	AGCCTCTTCCCAAATCTCAGC	AAAGCCTCCAACTCCGGACC
WWP1_P2	GCACCAATTTCAACTATGAAGCC	TCTCTCCCCTTCCATTTTAACTG
β-actin	CATGTACGTTGCTATCCAGGC	CTCCTTAATGTCACGCACGAT

**Table S3: The siRNA sequences.**		
Name	siRNA sequences - Sense (5'-3')	siRNA sequences - Anti-sense (5'-3')
si-NC	UUCUCCGAACGUGUCACGUTT	ACGUGACACGUUCGGAGAATT
si-E2F8 #1(sh-E2F8)	GGUUGUAUGAUAUAGCUAATT	UUAGCUAUAUCAUACAACCTT
si-E2F8 #2	GCCCUAUCAAGACCAACAATT	UUGUUGGUCUUGAUAGGGCTT
si-WWP1 #1(sh-WWP1)	GCUGCUCGUAUGUAGUUAATT	UUAACUACAUACGAGCAGCTT
si-WWP1 #2	GAGCUAUGCAACAGUUUAATT	UUAAACUGUUGCAUAGCUCTT

106
