# Supplementary material for: E2F-mediated activation of mTORC1 through the ubiquitin-proteasome system promotes lung adenocarcinoma progression
Source: Cell Death Dis. 2026 May 19;17(1):635. doi: 10.1038/s41419-026-08863-2 (PMC13358063; doi:10.1038/s41419-026-08863-2)

Fig 2 A

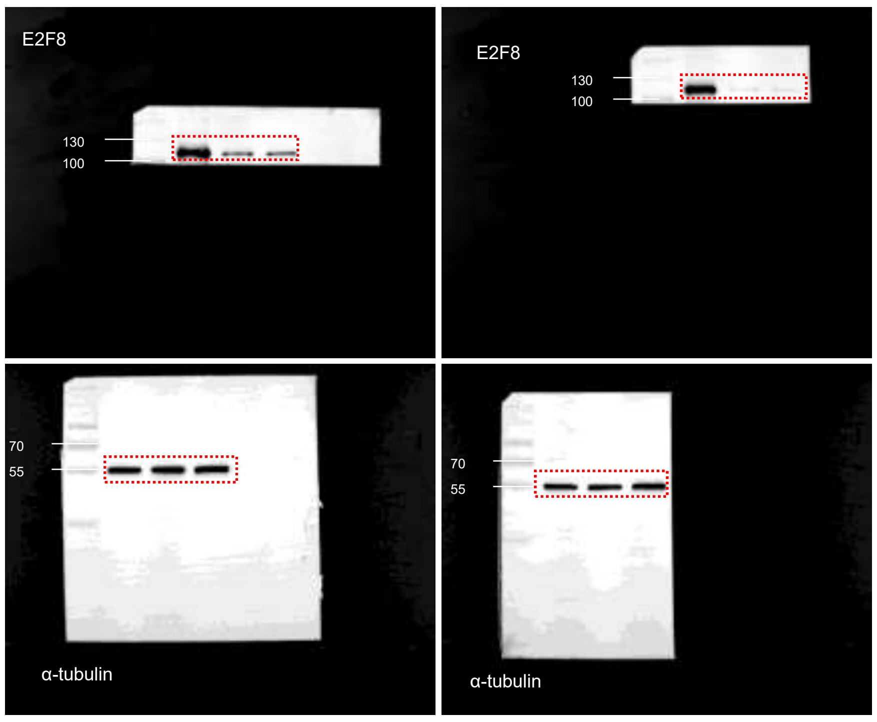

Fig 2 B

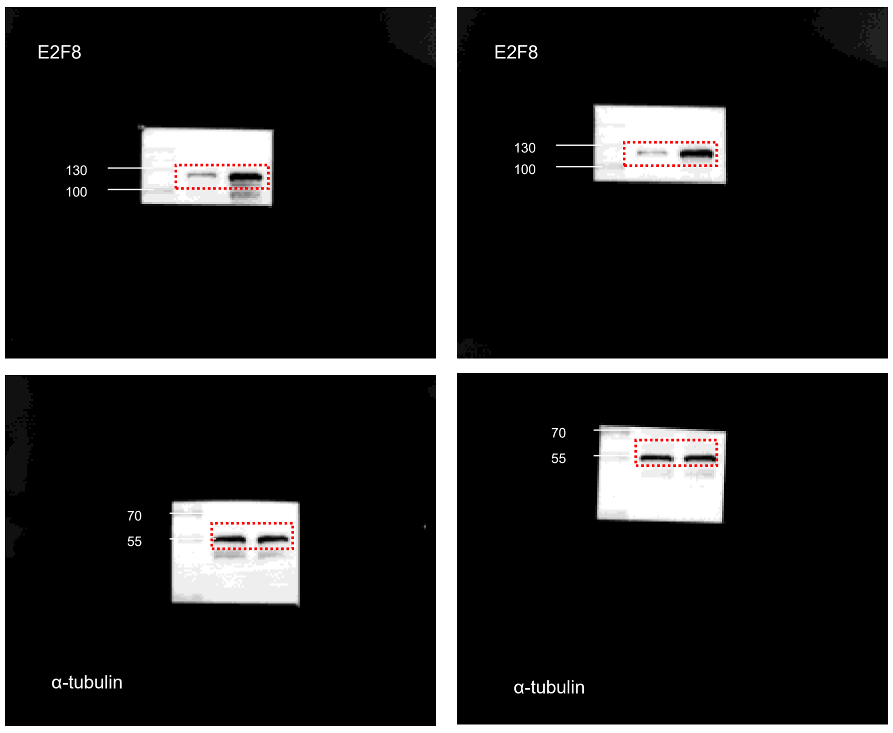

Fig 3 F

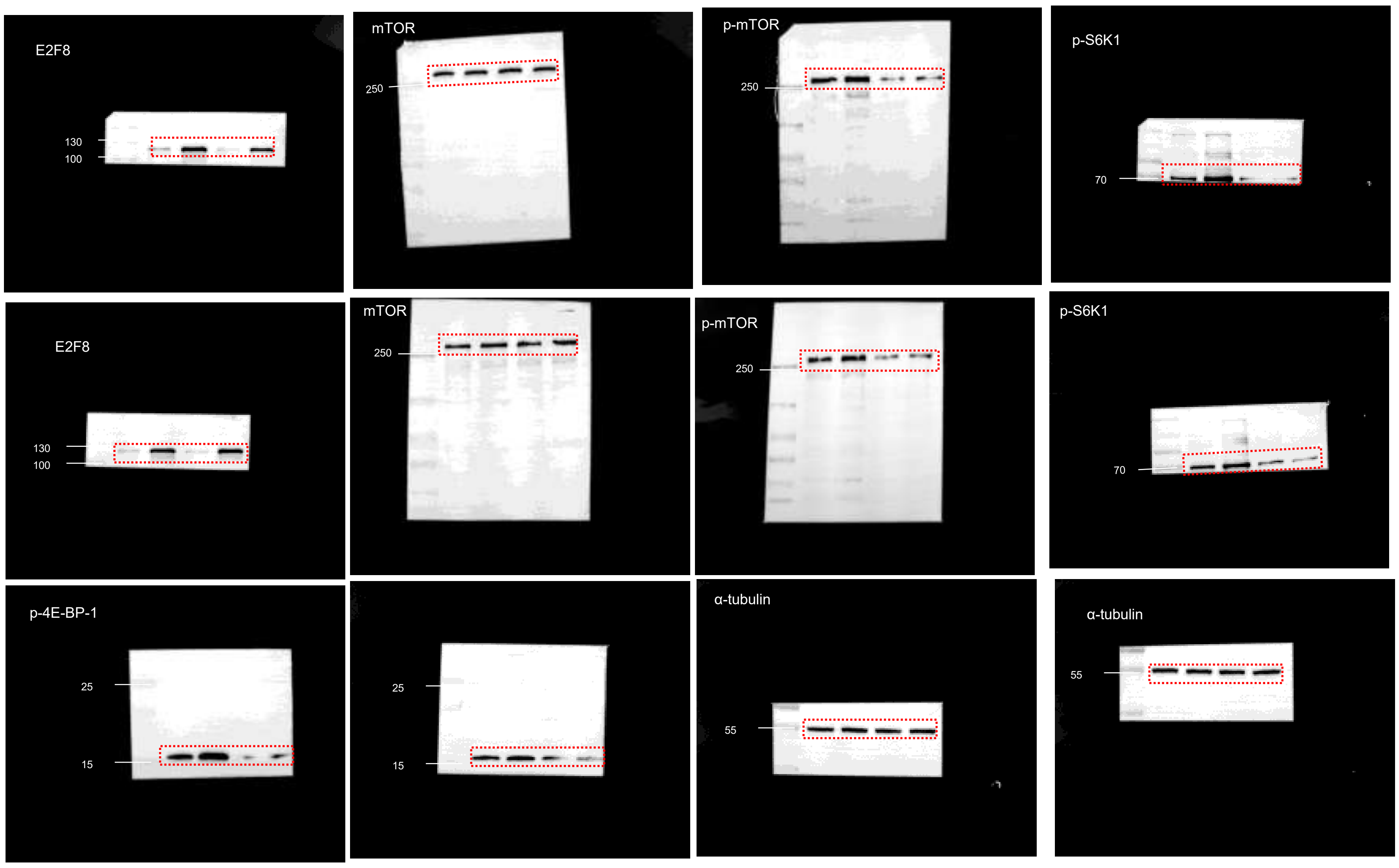

Fig 4 F

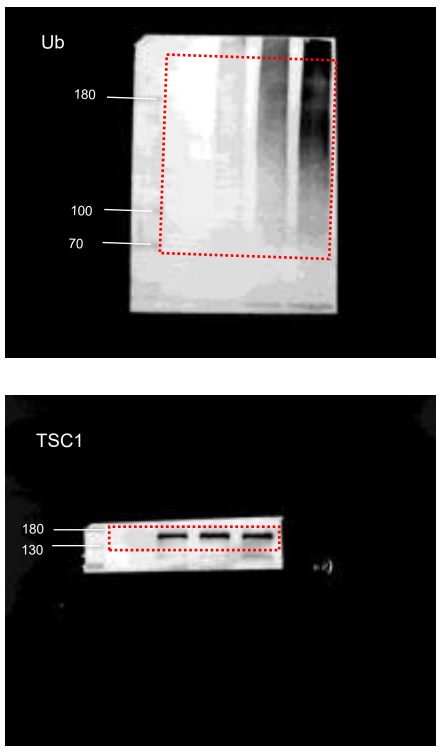

Fig 4 C

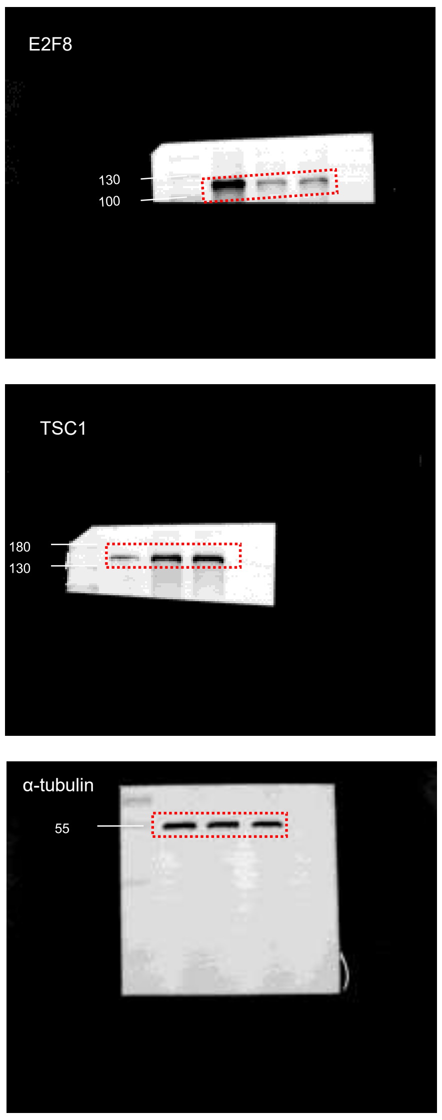

Fig 4 D

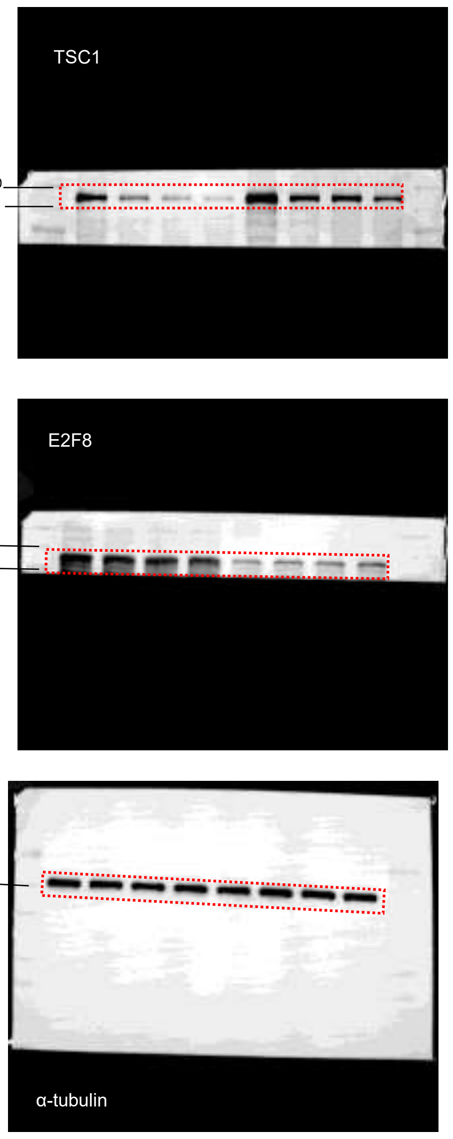

Fig 4 E

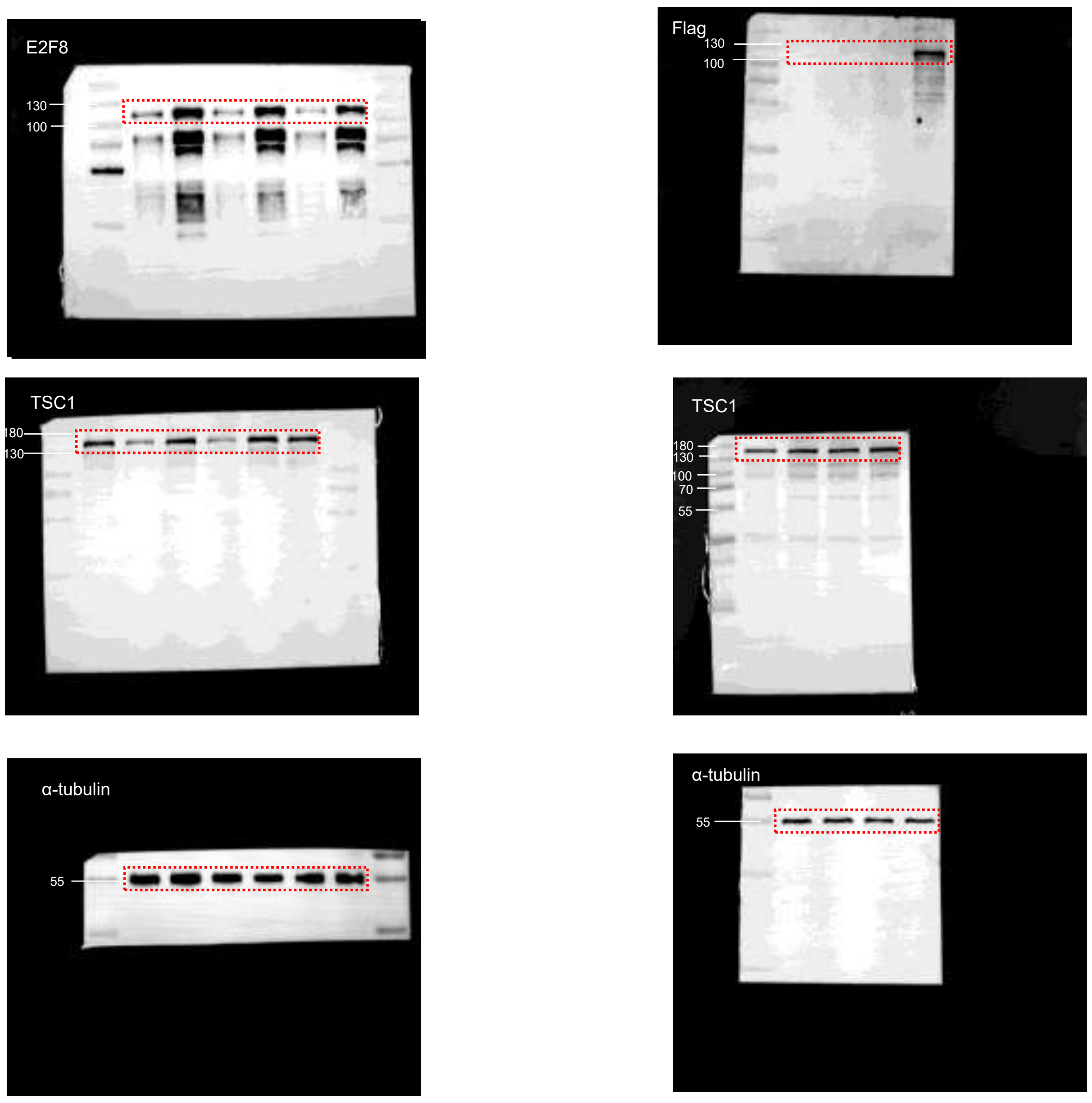

Fig 5 B

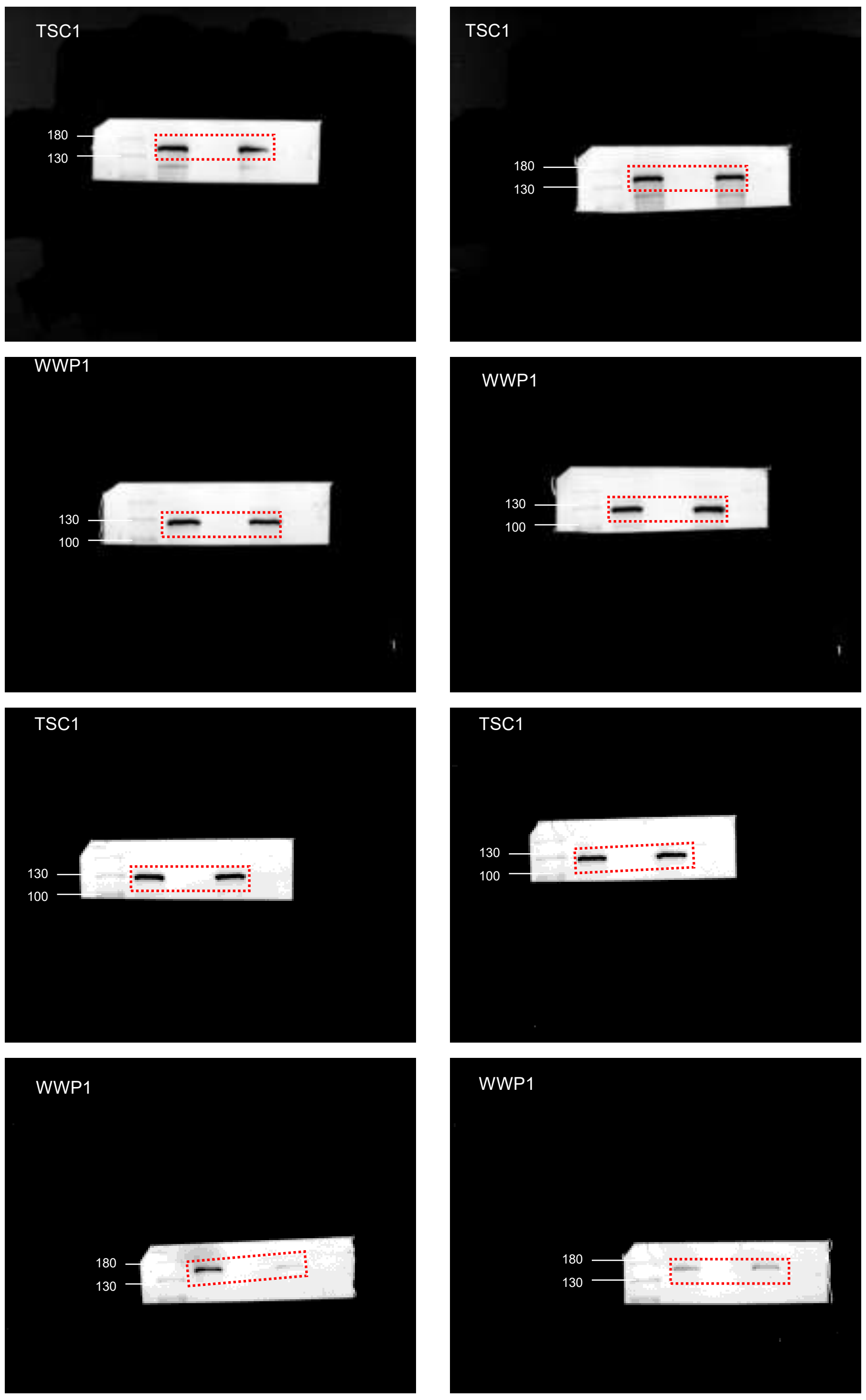

Fig 5 D

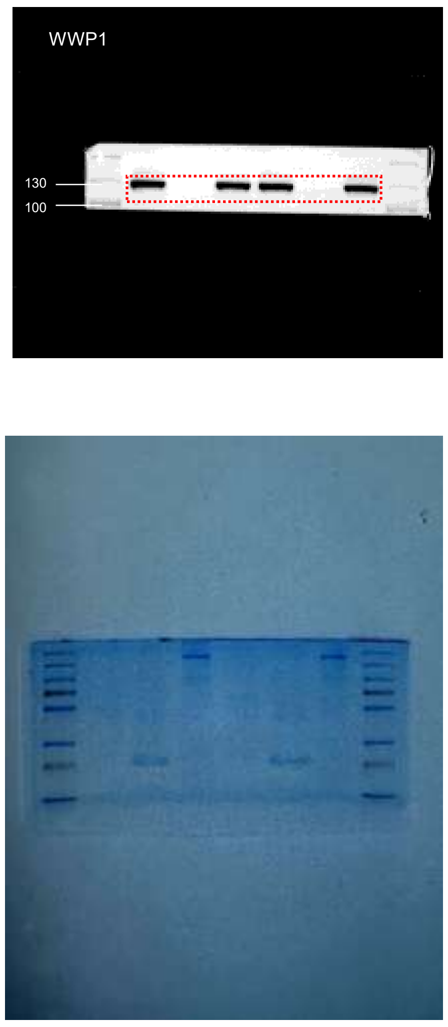

Fig 5 F

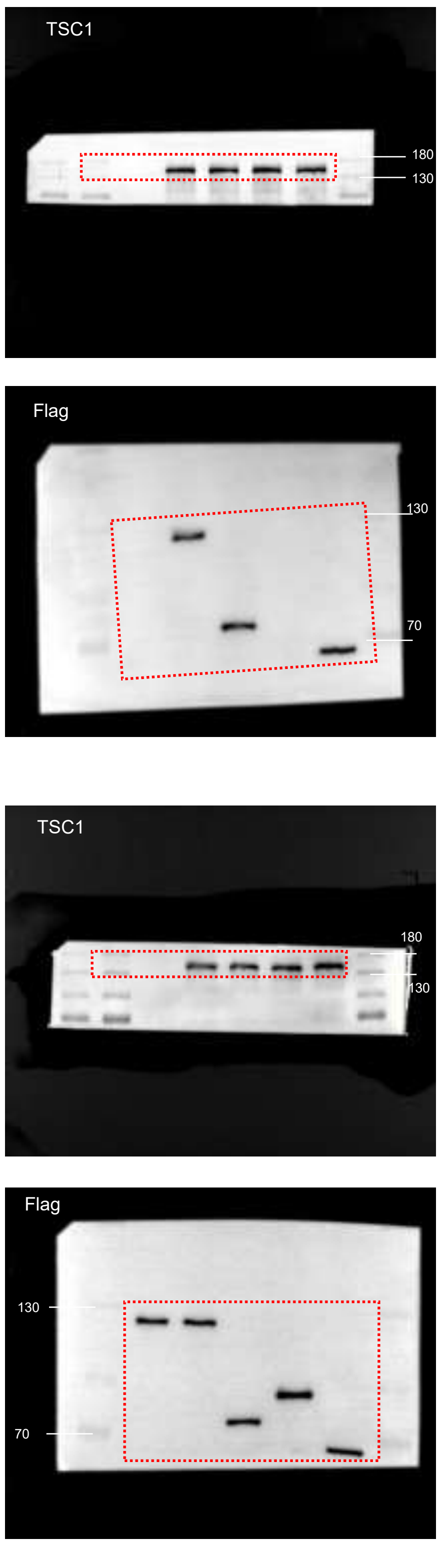

Fig 5 H

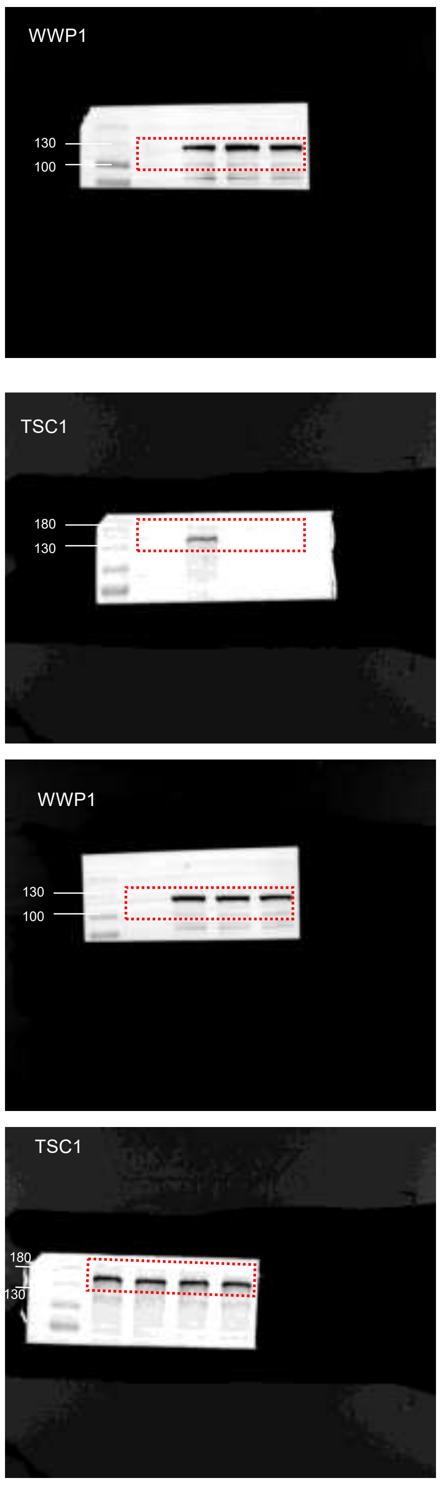

Fig 5 I

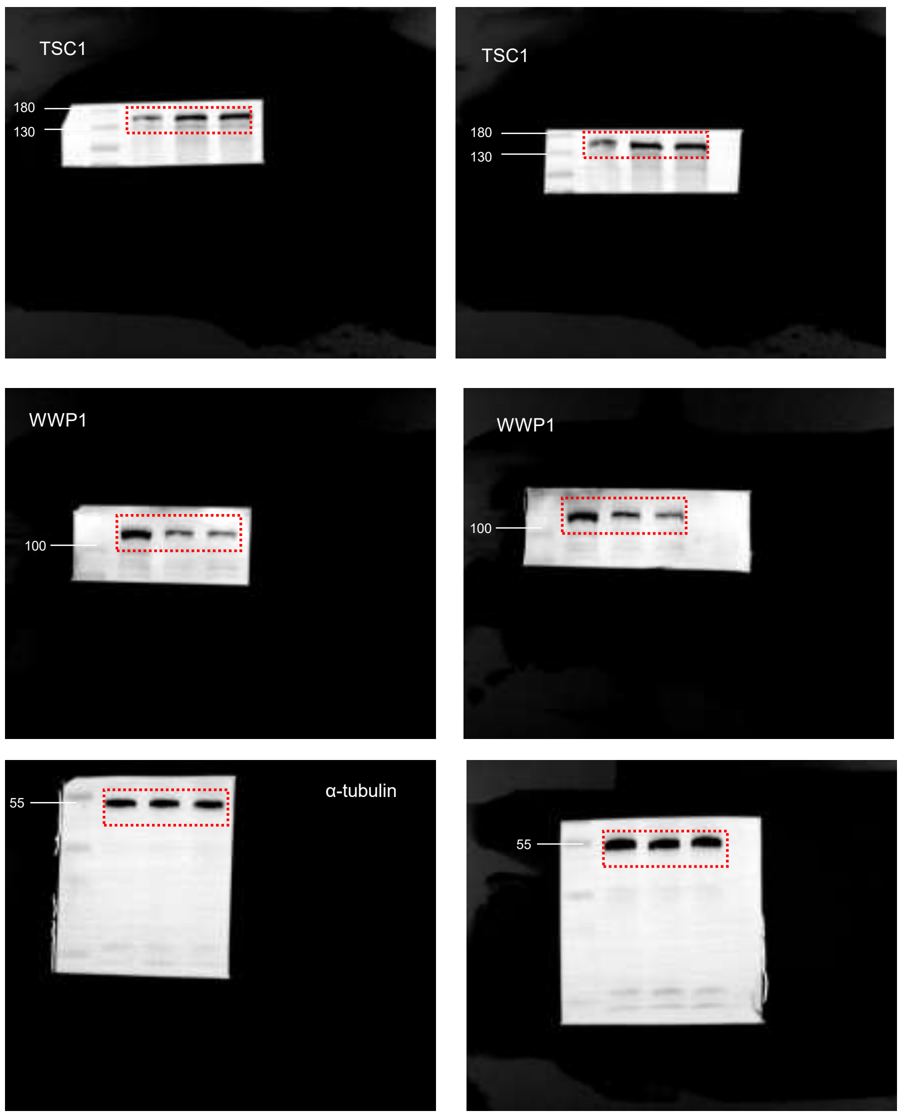

Fig 5 J

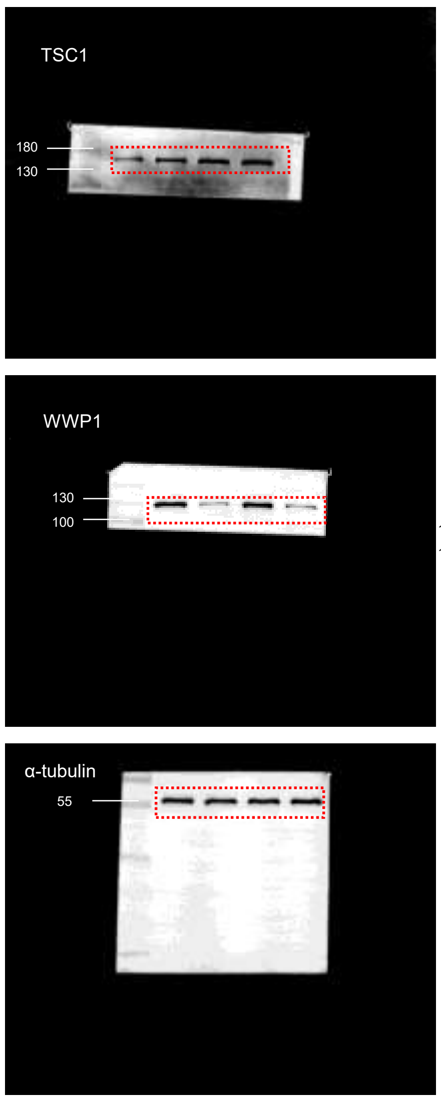

Fig 5 K

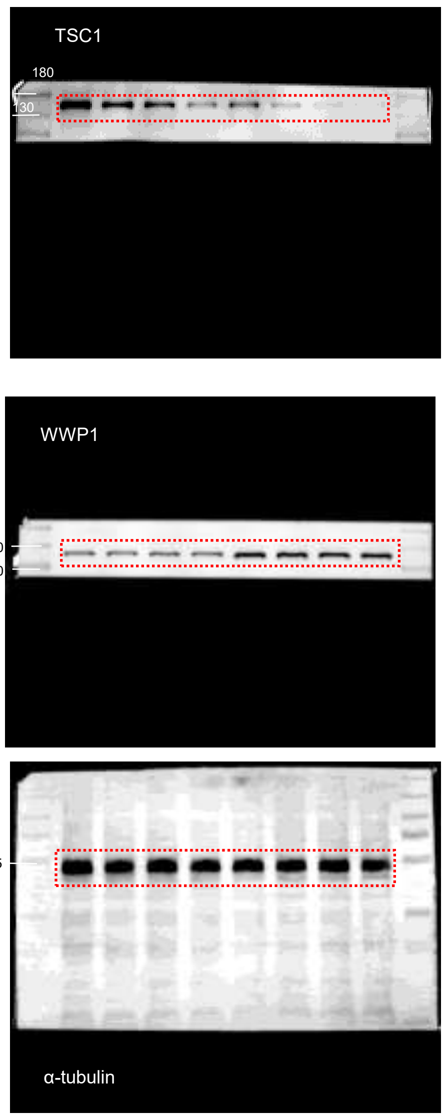

Fig 5 L

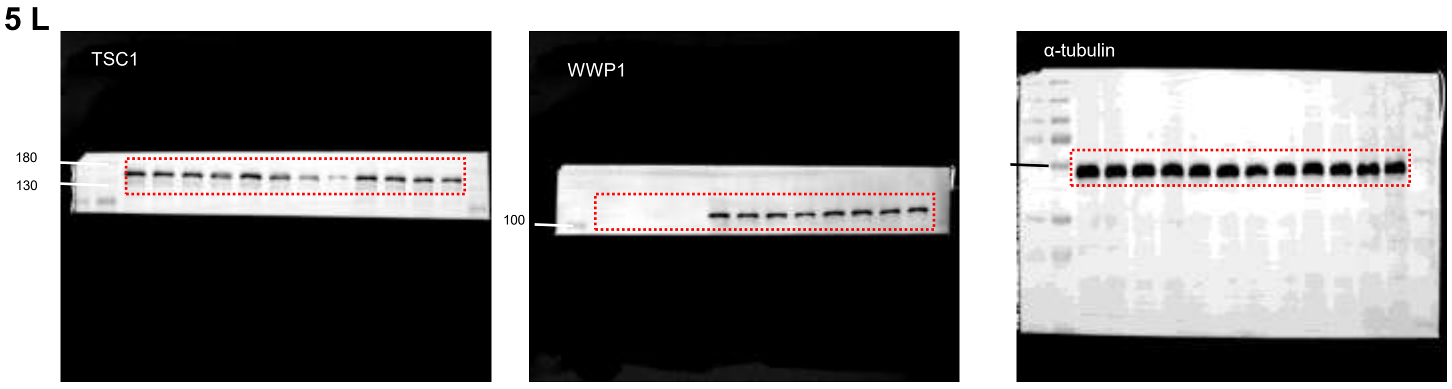

Fig 6 A

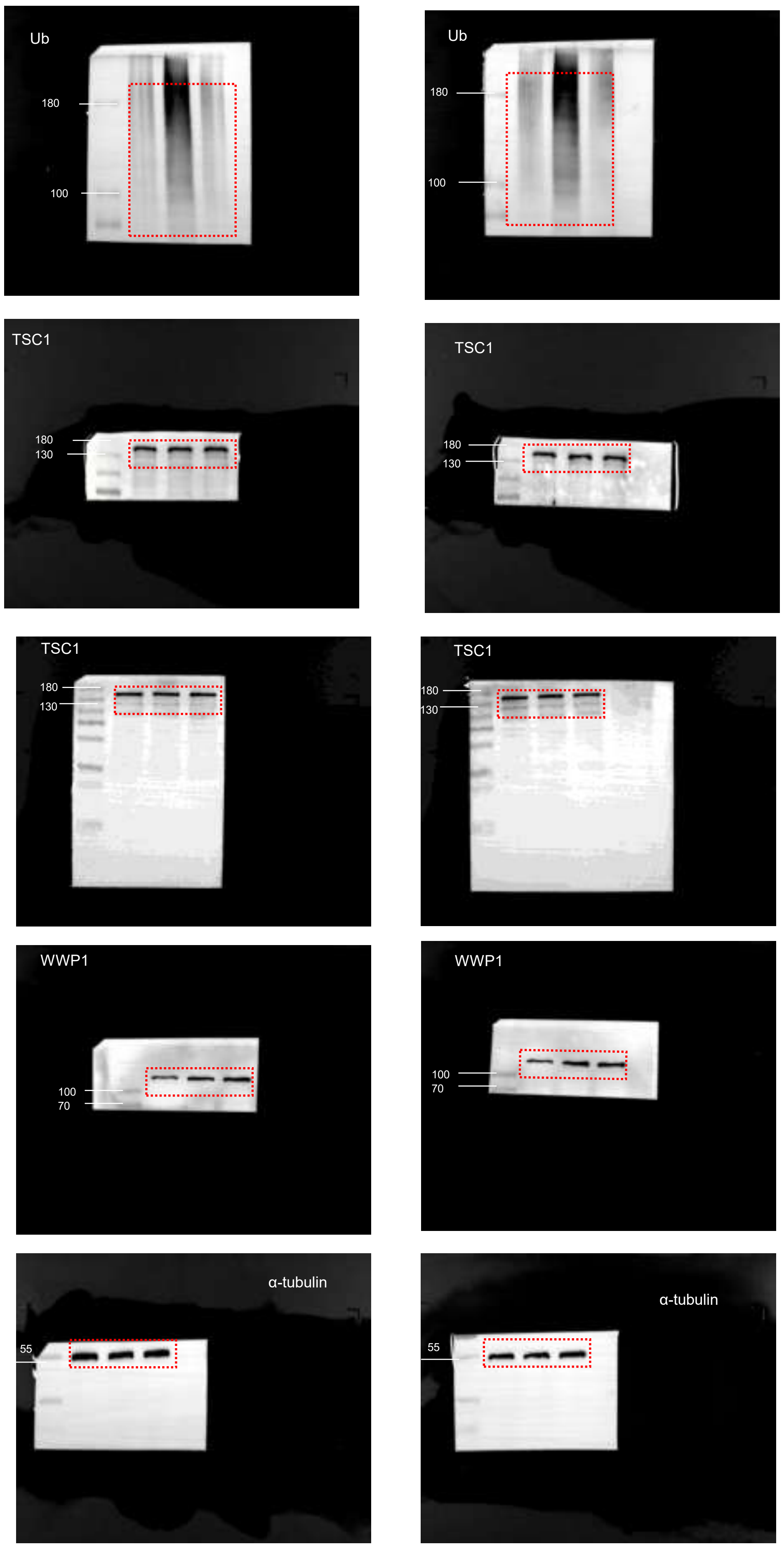

Fig 6 B

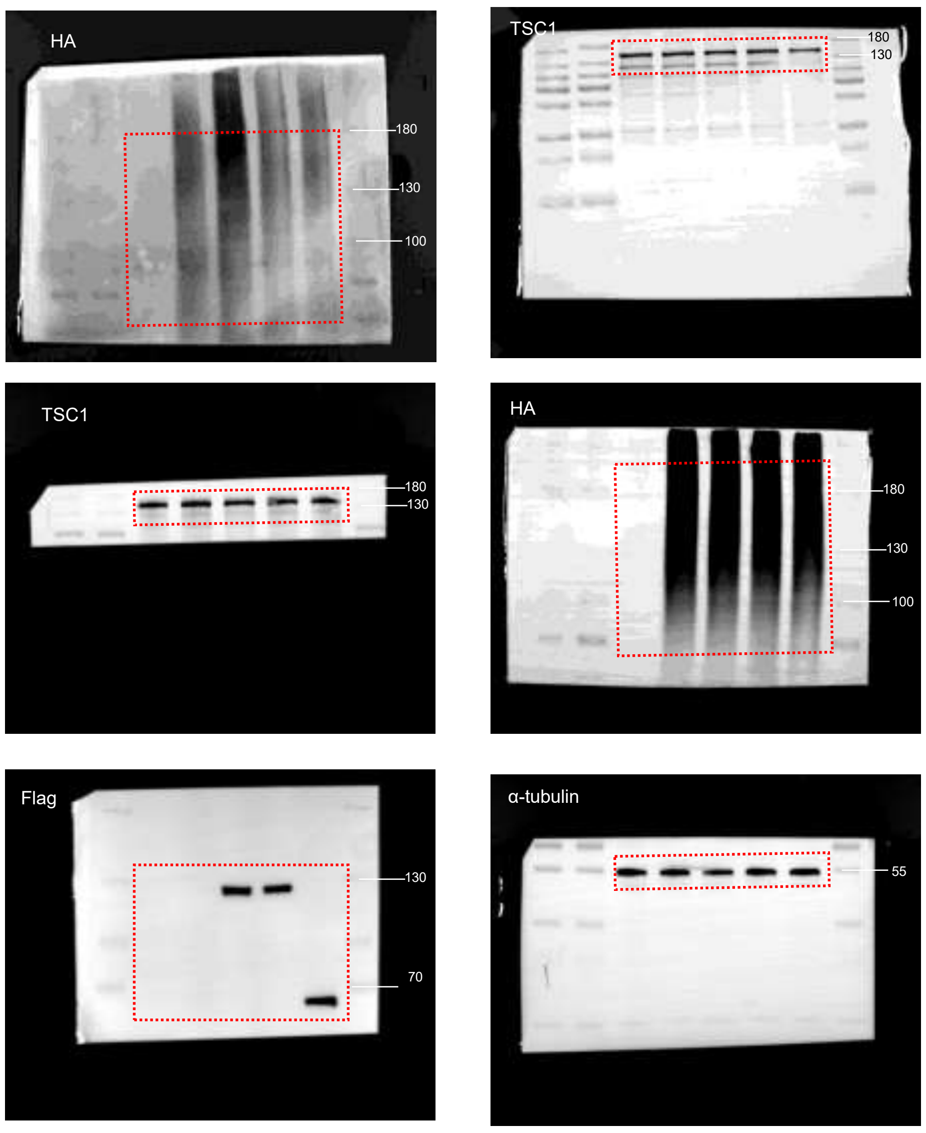

Fig 6 C

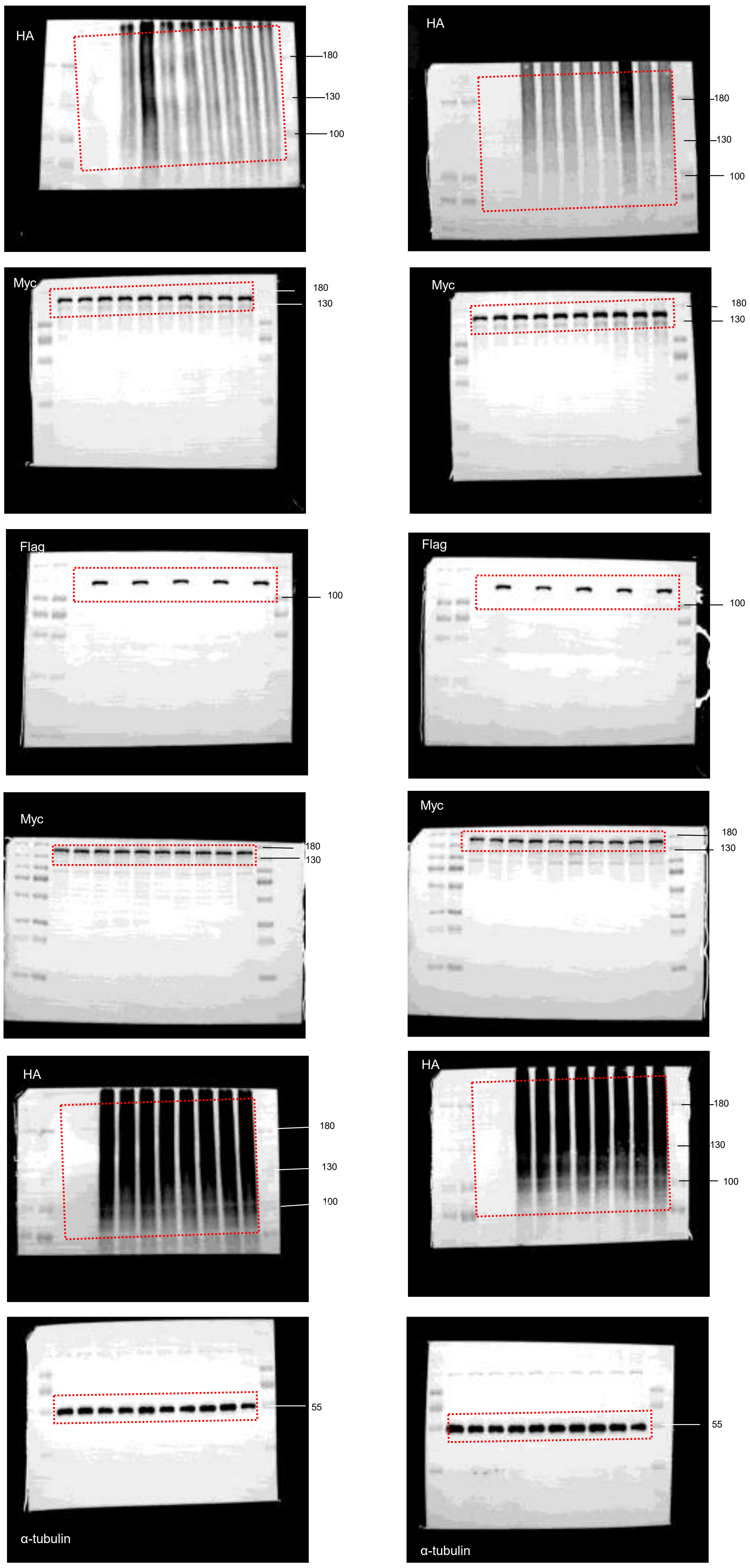

Fig 6 D

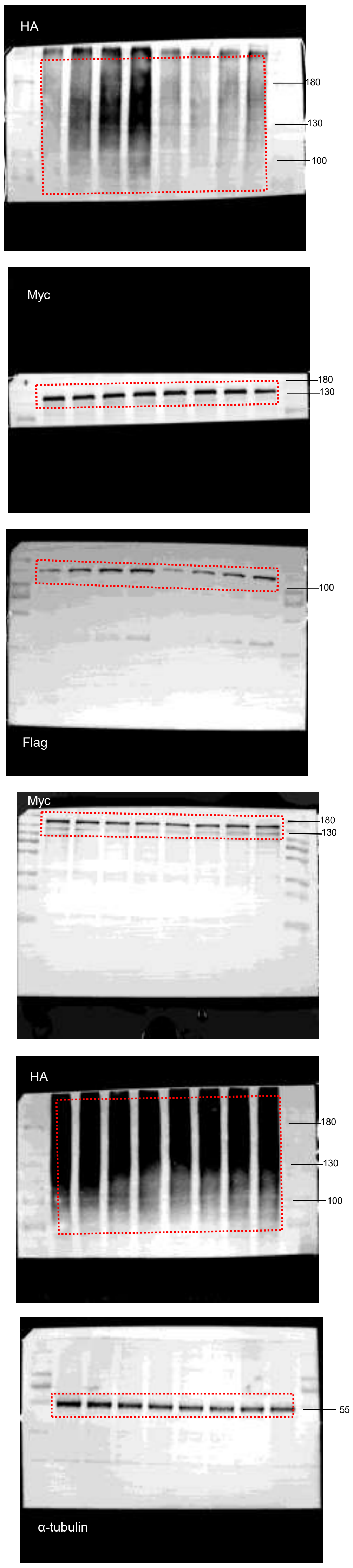

Fig 6 E

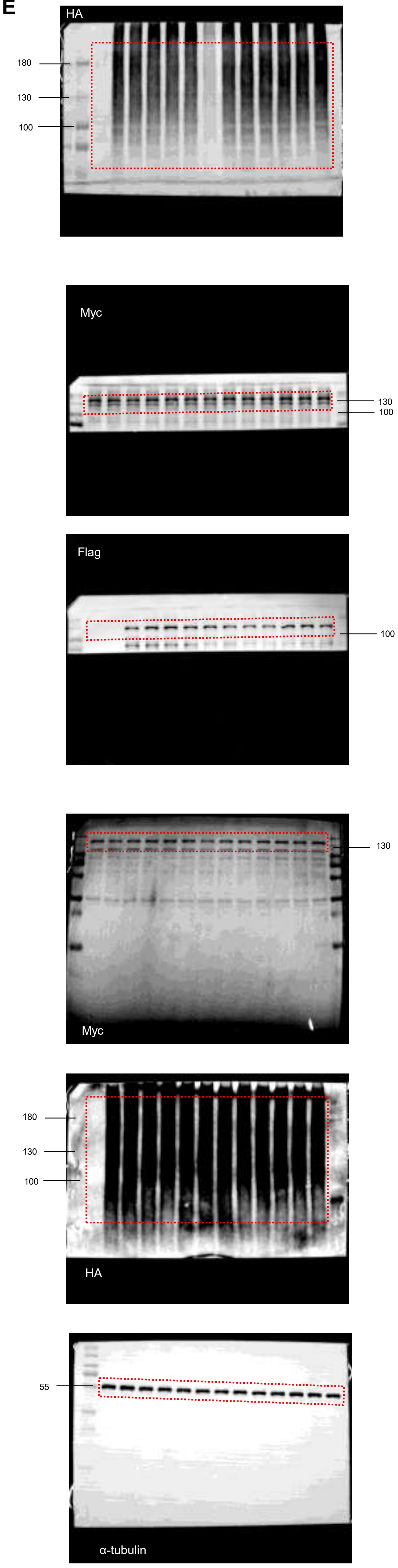

Fig 6 F

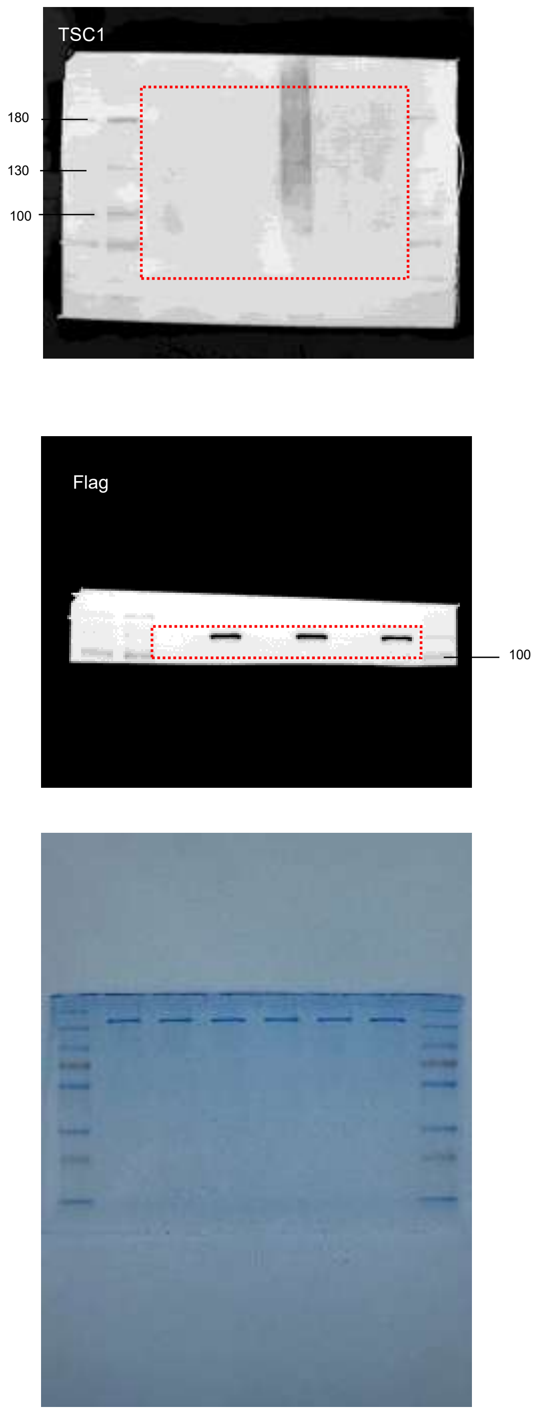

Fig S1 N

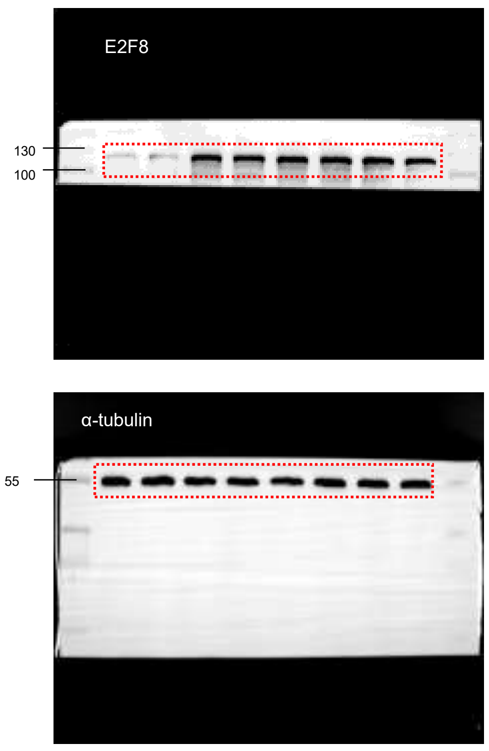

Fig S2 C

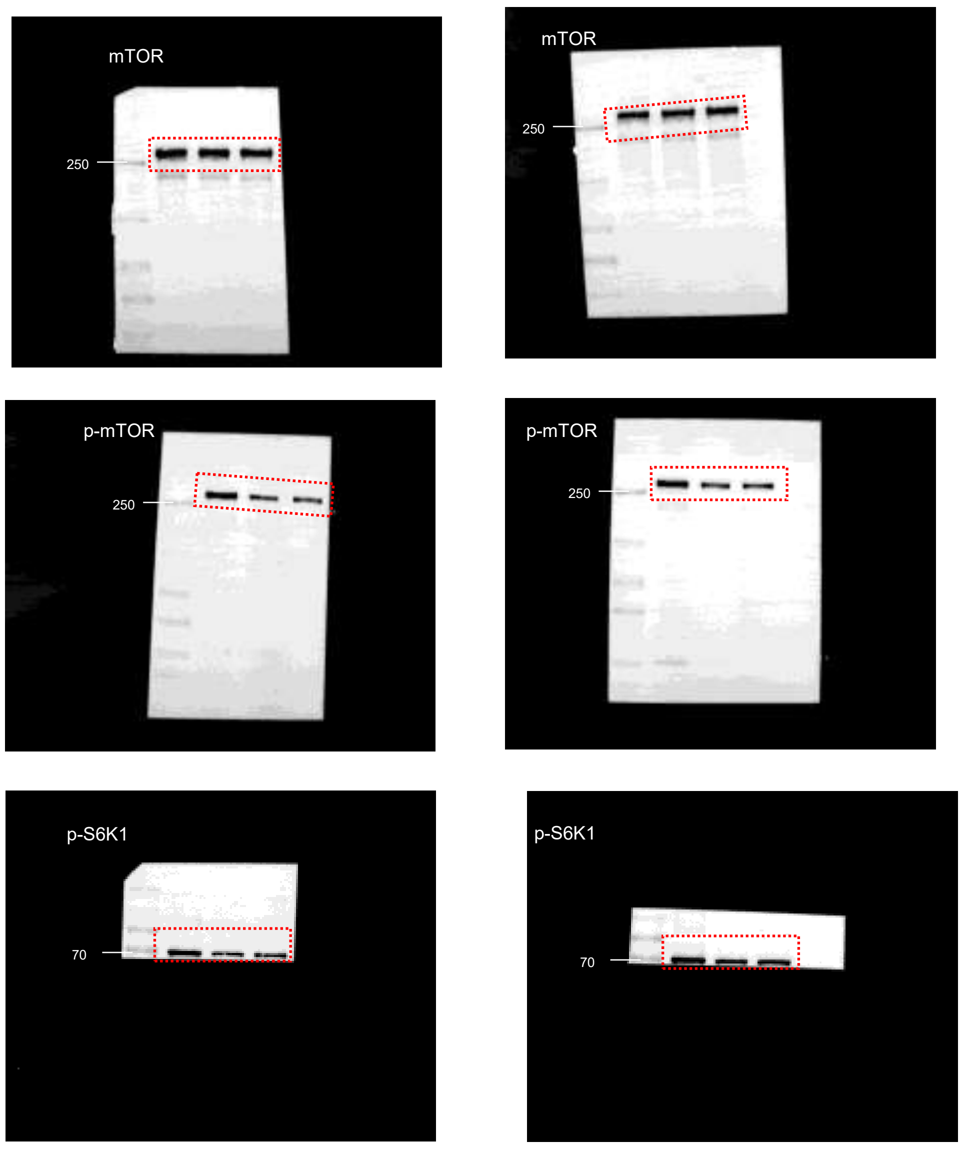

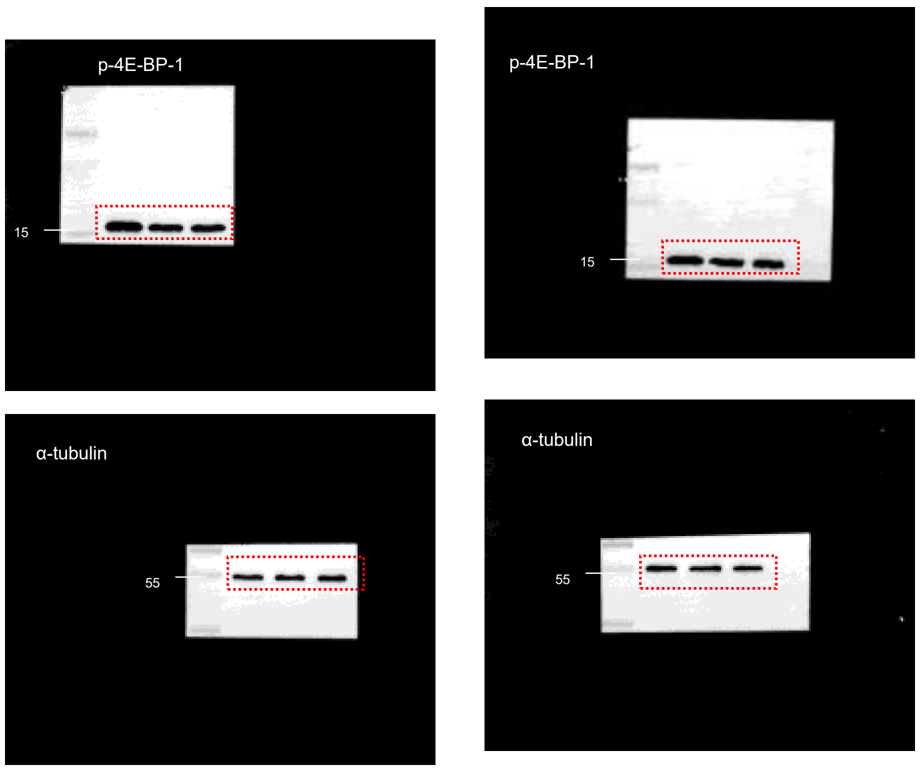

Fig S3 H

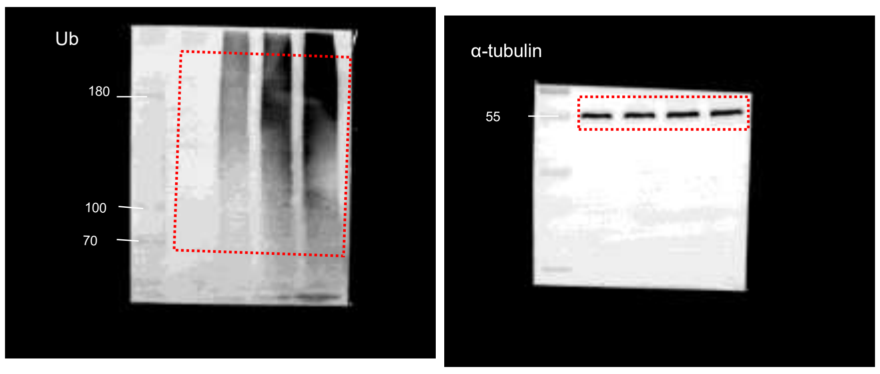

Fig S3 D

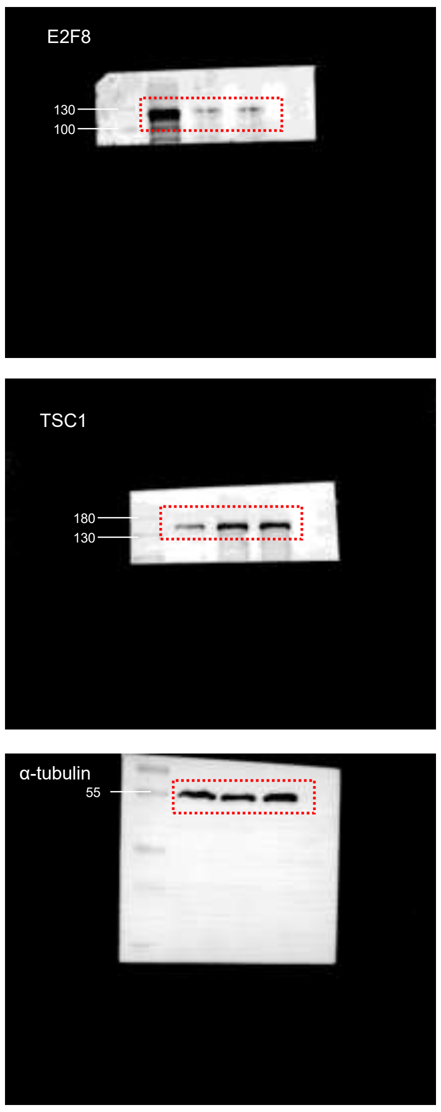

Fig S3 E

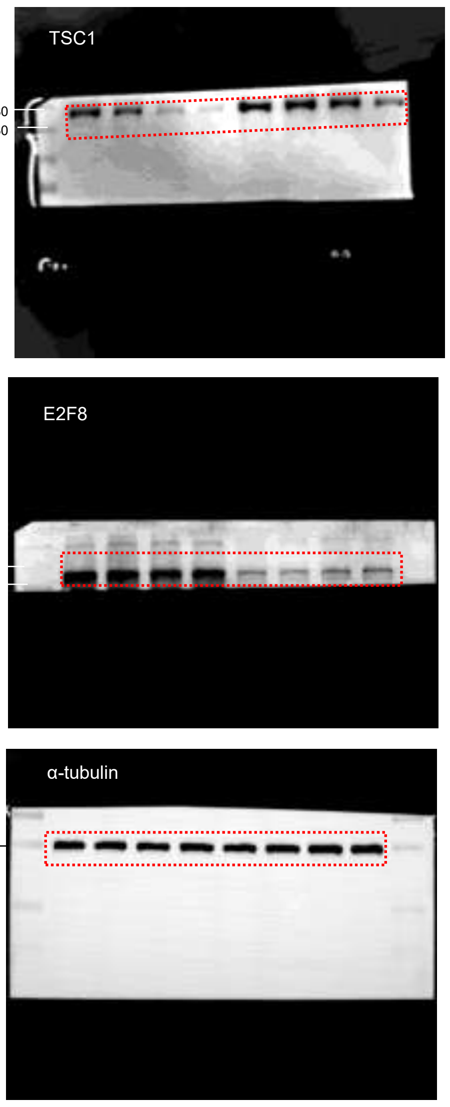

Fig S3F

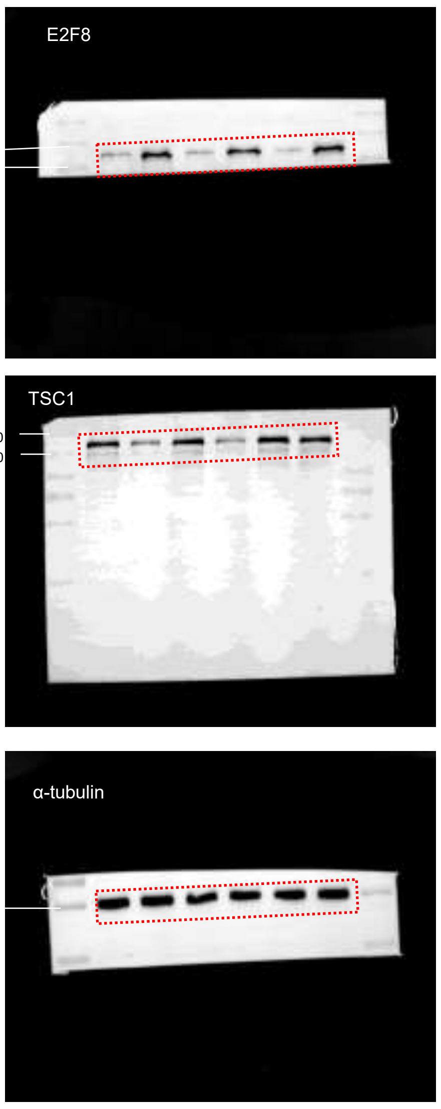

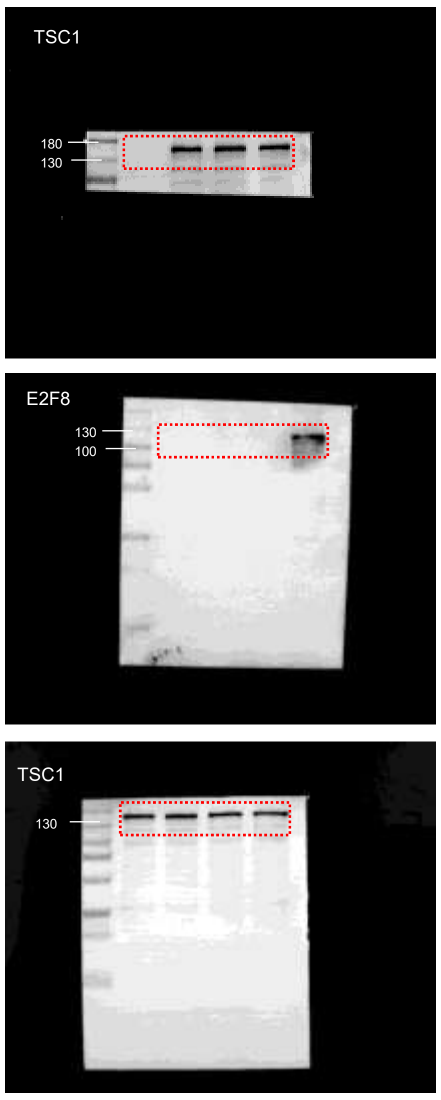

Fig S3-I-J

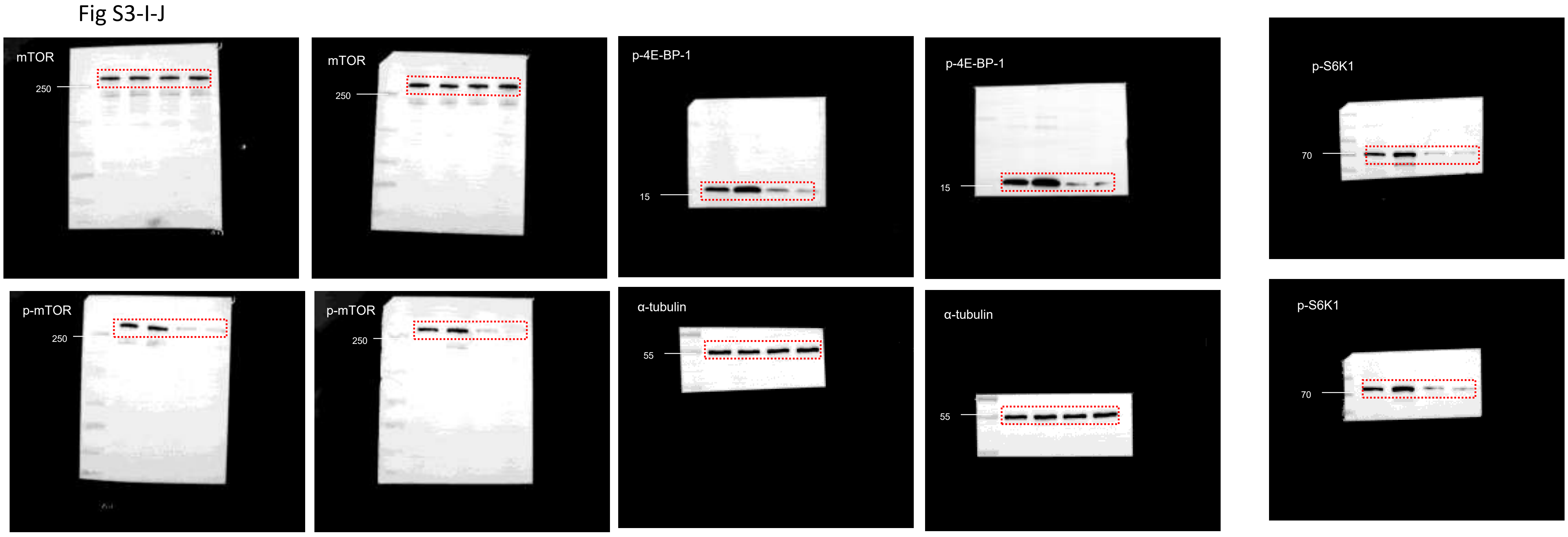

Fig S4-E

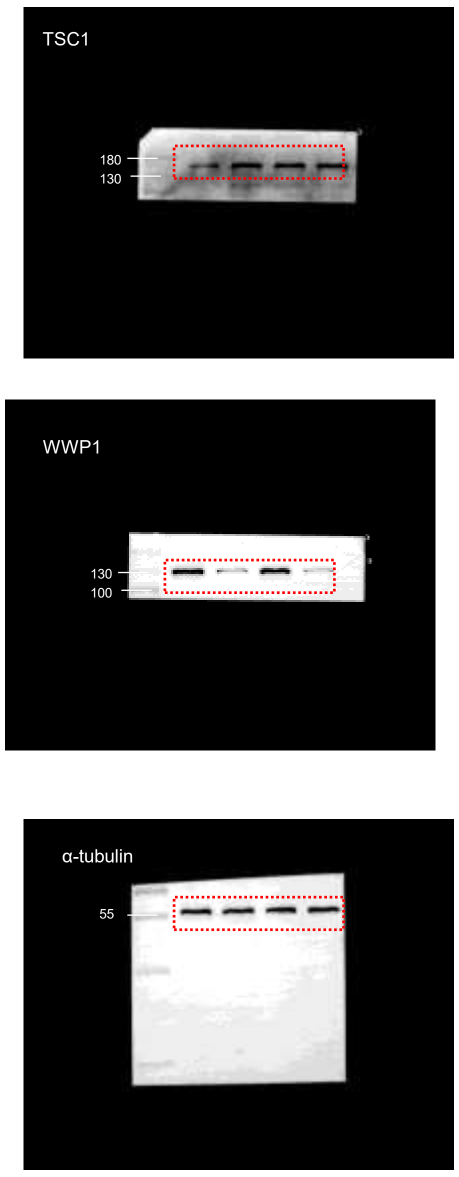

Fig S4-G

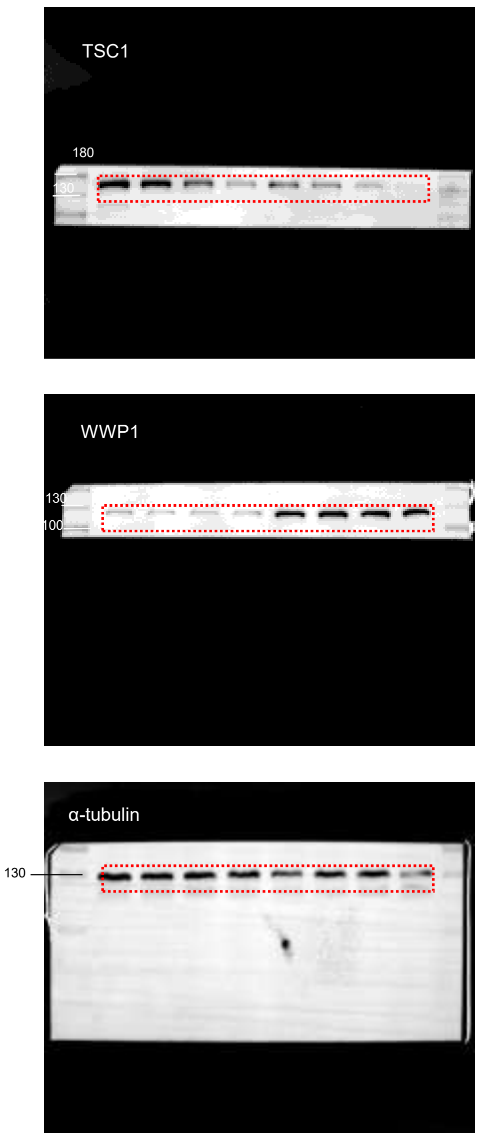

Fig S5-B

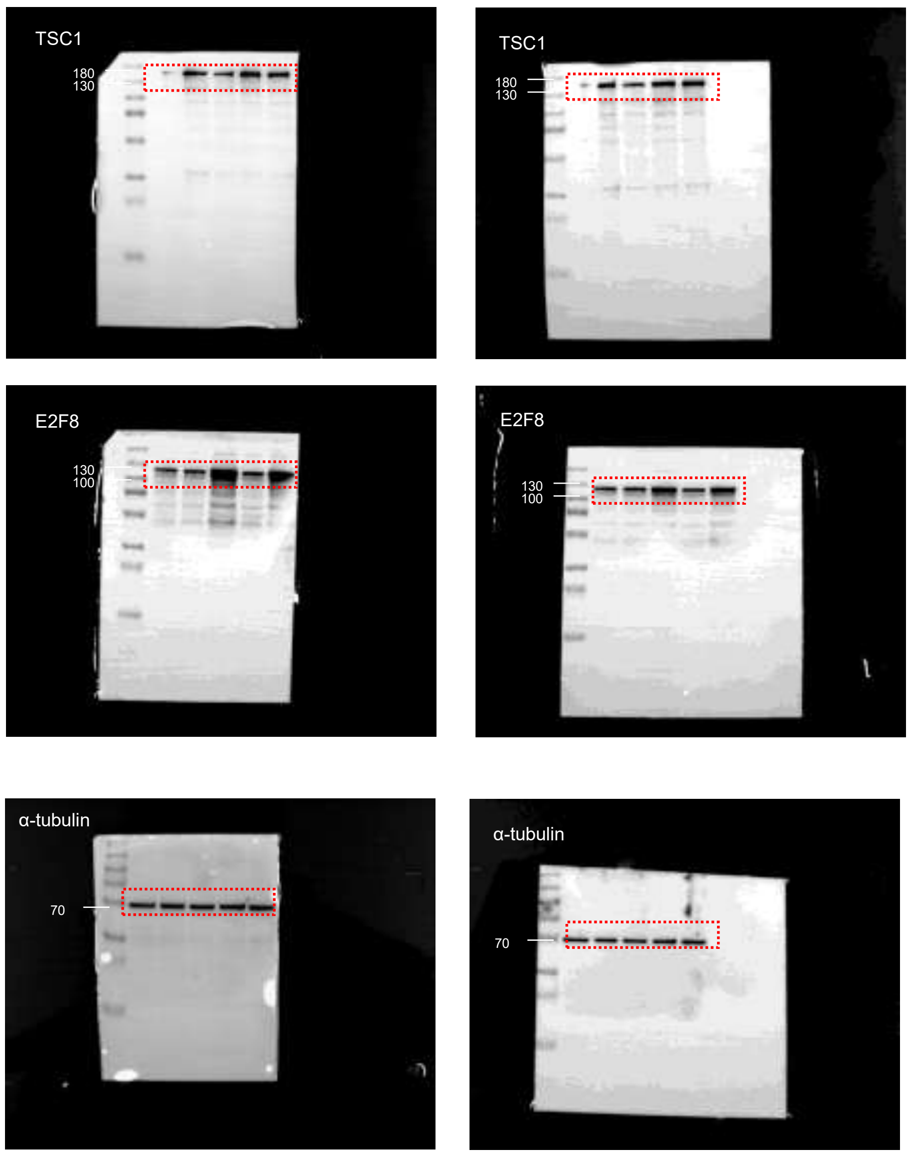

Fig S6E

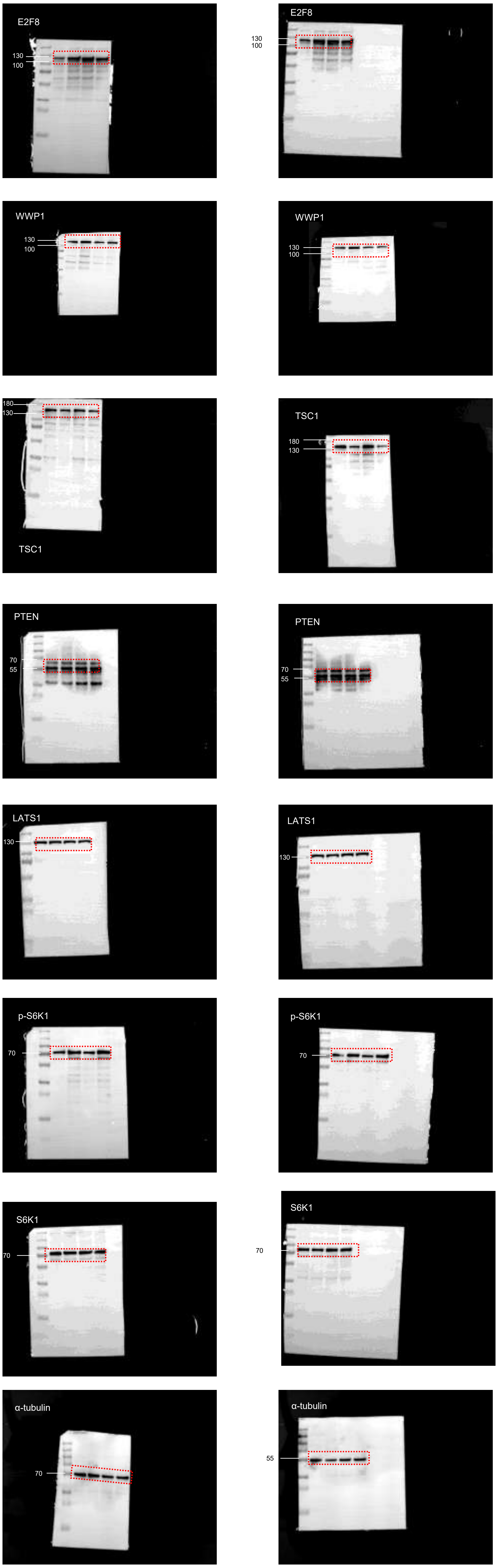

Supplement: Supplementary file 2 — Original western blots [file 41419_2026_8863_MOESM2_ESM.pdf]
